# The combinatorial control of alternative splicing in *C*. *elegans*

**DOI:** 10.1371/journal.pgen.1007033

**Published:** 2017-11-09

**Authors:** June H. Tan, Andrew G. Fraser

**Affiliations:** 1 The Donnelly Centre, University of Toronto, Toronto, ON, Canada; 2 Department of Molecular Genetics, University of Toronto, 1 King’s College Circle, Toronto, ON, Canada; Cold Spring Harbor Laboratory, UNITED STATES

## Abstract

Normal development requires the right splice variants to be made in the right tissues at the right time. The core splicing machinery is engaged in all splicing events, but which precise splice variant is made requires the choice between alternative splice sites—for this to occur, a set of splicing factors (SFs) must recognize and bind to short RNA motifs in the pre-mRNA. In *C*. *elegans*, there is known to be extensive variation in splicing patterns across development, but little is known about the targets of each SF or how multiple SFs combine to regulate splicing. Here we combine RNA-seq with *in vitro* binding assays to study how 4 different *C*. *elegans* SFs, ASD-1, FOX-1, MEC-8, and EXC-7, regulate splicing. The 4 SFs chosen all have well-characterised biology and well-studied loss-of-function genetic alleles, and all contain RRM domains. Intriguingly, while the SFs we examined have varied roles in *C*. *elegans* development, they show an unexpectedly high overlap in their targets. We also find that binding sites for these SFs occur on the same pre-mRNAs more frequently than expected suggesting extensive combinatorial control of splicing. We confirm that regulation of splicing by multiple SFs is often combinatorial and show that this is functionally significant. We also find that SFs appear to combine to affect splicing in two modes—they either bind in close proximity within the same intron or they appear to bind to separate regions of the intron in a conserved order. Finally, we find that the genes whose splicing are regulated by multiple SFs are highly enriched for genes involved in the cytoskeleton and in ion channels that are key for neurotransmission. Together, this shows that specific classes of genes have complex combinatorial regulation of splicing and that this combinatorial regulation is critical for normal development to occur.

## Author summary

Alternative splicing (AS) is a highly regulated process that is crucial for normal development. It requires the core splicing machinery, but the specific choice of splice site during AS is controlled by splicing factors (SFs) such as ELAV or RBFOX proteins that bind to specific sequences in pre-mRNAs to regulate usage of different splice sites. AS varies across the *C*. *elegans* life cycle and here we study how diverse SFs combine to regulate AS during *C*. *elegans* development. We selected 4 RRM-containing SFs that are all well studied and that have well-characterised loss-of-function genetic alleles. We find that these SFs regulate many of the same targets, and that combinatorial interactions between these SFs affect both individual splicing events and organism-level phenotypes including specific effects on the neuromuscular system. We further show that SFs combine to regulate splicing of an individual pre-mRNA in two distinct modes—either by binding in close proximity or by binding in a defined order on the pre-mRNA. Finally, we find that the genes whose splicing are most likely to be regulated by multiple SFs are genes that are required for the proper function of the neuromuscular system. These genes are also most likely to have changing AS patterns across development, suggesting that their splicing regulation is highly complex and developmentally regulated. Taken together, our data show that the precise splice variant expressed at any point in development is often the outcome of regulation by multiple SFs.

## Introduction

Alternative splicing (AS) is highly regulated. Many genes have different splice patterns in different tissues and at different developmental stages, and splicing can also change in response to external cues (reviewed in [1]). AS plays a crucial role in the proper development of all animals [2–7] and AS is typically widely used to generate proteome diversity. For example, in humans ~95% of multi-exon genes are estimated to be alternatively spliced [8,9] and errors in regulation of AS can lead to a variety of human diseases, ranging from muscular dystrophy to cystic fibrosis to various neurological disorders [10].

Splicing requires the core splicing machinery but for the correct choice of splice site, specific regulatory splicing factors (we refer to these throughout as SFs) recognize short *cis*-regulatory elements in the pre-mRNA [11]—these SFs can either select for or repress the use of any specific exon-exon junction (reviewed in [12–14]). The precise combination of SFs that bind any particular pre-mRNA thus determine which exon-exon junctions are selected and hence which mature mRNA is made [15,16]. This is complex—each cell type expresses many different SFs and introns frequently contain binding sites for many SFs. To understand how AS is regulated thus requires us to know not only how individual SFs recognize and regulate any splice event but also how multiple SFs combine to affect splicing. Addressing this combinatorial regulation of splicing is central to the work we present here.

Several genomics technologies have transformed our ability to identify splice variants and this has given major recent insights into splicing regulation. In particular RNA-seq is an extremely powerful tool for identifying the splice variants that are present in any tissue or cell type and splicing profiles have been generated for many cell-types [17–20]. Other high-throughput technologies have also allowed the genome-scale characterization of the *cis*- and *trans*-acting factors involved in regulating AS events [13,21,22] and together these data have been used to define a regulatory ‘splicing code’ of RNA features that predict splicing patterns [16,23,24]. In addition, studies on individual SFs have resulted in RNA splicing maps [25] for several SFs, including Nova [26,27], RBFOX [28–30], PTB [31–33], hnRNP [34,35], TDP-43 [36], and TIA [37] proteins, which provide mechanistic insights into how binding positions correlate to regulatory effects on splicing [23,38,39].

Almost all these studies were carried out on individual cell-lines or isolated tissues and typically present a description of the splicing pattern in any specific cell-type—which isoforms are expressed and which SFs regulate these. During the development of an animal from one cell to the mature adult, splicing patterns change greatly however and this dynamic AS must be highly regulated and orchestrated. To begin to understand how AS regulation is coordinated across animal development, we use *C*. *elegans* as a simple animal model (its use as a model for studying AS regulation is reviewed in [3,40]). *C*. *elegans* development is well-characterized and its lineage is identical in every animal [41,42]. The overall splicing machinery in *C*. *elegans* is conserved with humans [43], and there is extensive AS: ~25% of genes are estimated to be alternatively spliced and many AS events show clear developmental regulation [44]. In *C*. *elegans*, key studies have demonstrated intricate co-regulation of splicing by multiple SFs [45–50], from examples of cooperative regulation (e.g. for ASD-1/FOX1 and SUP-12 [46,51]), to examples of tissue-specific splicing driven by differential tissue expression of SFs such as AS regulation by EXC-7 and UNC-75 [48]. In addition, high-throughput studies have shown that many AS events are co-regulated by multiple diverse SFs [52]. However, the *cis*- and *trans*- factors that regulate AS in the worm are underexplored—in particular only a handful of *C*. *elegans* SF targets are known. As a first step towards mapping the networks that regulate AS across *C*. *elegans* development, we thus aim to systematically identify targets that are regulated by each SF. In this initial study, we principally focus on 4 different SFs—ASD-1, FOX-1, EXC-7 and MEC-8.

**Fig 1:**
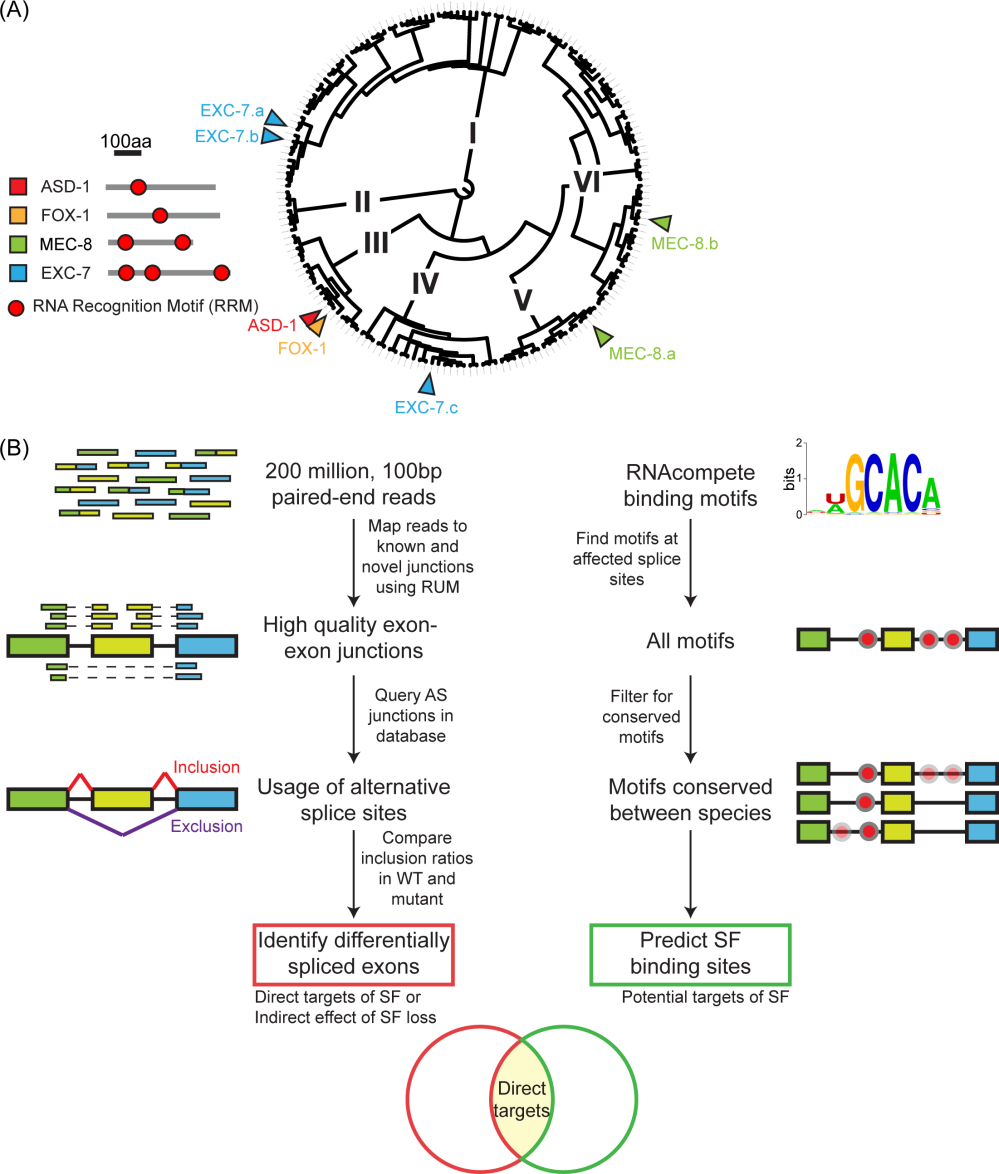
Predicting direct targets of 4 diverse SFs. (A) The SFs in this study all contain one or more RRM domain(s). In comparison to all RRM domains encoded in the *C*. *elegans* genome, the RRMs in these SFs fall into distinct clades based on sequence similarity. The scale bar refers to the length of SF proteins. (B) Direct targets of each of the 4 SFs are predicted by identifying differentially spliced exons by RNA-seq and querying surrounding sequences for presence of conserved SF binding motifs.

Each of the SFs we chose to study is well-characterised—their involvement in *C*. *elegans* development and function is known, their expression patterns well described, and they all contain RRM domains that are one of the best understood RNA binding domains that appear frequently in SFs involved in AS regulation [53]. There are 183 RRM domains encoded in the *C*. *elegans* genome [54,55]–found within 105 genes–and they belong to distinct subfamilies. The SFs that we have selected here have RRMs that cover 5 of 6 distinct clades (Fig 1A). FOX-1 is a member of the RBFOX family of SFs [56]. It is involved in sexual differentiation by regulating splicing of *xol-1* [57] and also functions to regulate splicing of a fibroblast growth factor receptor gene, *egl-15* [45]. ASD-1 is a paralog of FOX-1, and was identified in a screen for other *egl-15* splicing regulators [45], and redundantly regulates *egl-15* splicing along with FOX-1. Both ASD-1 and FOX-1 are widely expressed in the neuromuscular system [45]. MEC-8 is a nuclear protein known to regulate alternative splicing of the *unc-52*, *mec-2*, and *fbn-1* transcripts [58–61], and mutations in *mec-8* leads to mechanosensory and chemosensory defects [62–64] as well as muscle defects. *mec-*8 is widely expressed in many tissues including both neurons and muscle cells in early development and becomes more restricted during development until it is confined to the six touch cells in later stages [59]. EXC-7 is a homolog of the *Drosophila* ELAV SF and is known to be involved in excretory cell formation [65], synaptic transmission [66] and neuron-specific AS events [48]. *exc-7* is expressed in a number of tissues including the excretory cell and is widely expressed in neurons [65,67]. Our goal is both to identify the targets of each individual SF, but also to examine how multiple SFs might combine to affect splicing across development.

Using RNA-seq, we identified AS events that are affected by loss of each SF *in vivo*. Coupled with data on RNA-binding specificities of these SFs [68], we identify the AS events that are likely to be direct targets of each SF by querying for the presence of a conserved SF RNA-binding motif. Using these data, we find strong evidence for combinatorial regulation of AS by these SFs and identify different ways in which the SFs appear to combine to affect AS events. Finally, we find that multiple of these SFs appear to co-regulate the splicing of genes with similar functions, including genes with key neuronal functions such as ligand-gated ion channels and cytoskeleton-binding proteins.

## Results

In this study, we wanted to assess how multiple SFs regulate splicing *in vivo* in *C*. *elegans*. To do this, we combined two main approaches. First, we used RNA-seq to identify the AS events that are affected by loss of individual SFs. RNA-seq is a powerful method to survey splice variants [69,70]. Comparing the splicing pattern seen in wild-type animal with that in a strain containing a loss-of-function mutation in a specific SF identifies the splicing events that are affected by the activity of that SF. Some of these are direct targets of that SF; others will be indirect downstream consequences of loss of that SF. To distinguish between these, we used the experimentally-determined binding specificities of these SFs: direct targets of any SF contain binding sites for that SF in their pre-mRNAs whereas indirect targets do not. This approach has previously been shown to accurately identify direct targets of SFs in *C*. *elegans* [46,48,49] and in cell lines [27,30,71,72], and found to be predictive in identifying sequences that are bound by various RNA-binding proteins *in vivo* [68]. Thus for each studied SF, we combine the *in vivo* effects of loss of that SF on splicing patterns with the known *in vitro* binding specificity for that SF in order to identify likely direct targets of that SF. This is illustrated schematically in Fig 1B and provides a starting point to examine how multiple SFs combine to affect splicing *in vivo*.

### Systematic identification of differentially regulated splice sites in SF mutants

We used RNA-seq to identify AS events that are affected by loss of each of 4 different SFs—ASD-1, FOX-1, MEC-8, and EXC-7. Each of these SFs is well-studied, there are well-characterized loss-of-function mutant alleles for each SF, and the *in vivo* functions of these SFs are known [63,65,73,74].

We sequenced polyA+ RNA isolated from wild-type and mutant worms harvested at the L4 developmental stage, obtaining approximately 200 million 100 bp, paired-end reads for each sample. Previous studies suggest that this read depth identifies the great majority of all splice variants present in the developing animal at this stage [44] and we observe similar results here (S1 Table). For each SF mutant, we identified differentially spliced exons (see Methods) that fall into several categories comprising major types of AS events (Table 1, S2 Table), such as cassette exons, alternative 5’ or 3’ splice sites, and mutually exclusive exons. We chose the L4 stage since both somatic tissues and germline are almost fully developed but, unlike adult samples, there is no risk of contamination with any fertilized embryos which can greatly affect transcriptomes. We also chose to examine the same developmental stage for all mutants so that we could easily compare any splice changes between mutants.

**Table 1:** Identification of differentially spliced exons in various mutant strains.

	Exon skipping		Multiple skipping		Alternative 3' SS		Alternative 5' SS		Mutually Exclusive	
Mutant	Annotated	Novel	Annotated	Novel	Annotated	Novel	Annotated	Novel	Annotated	Novel
*fox-1(e2643)*	6 (292^a^)	11	0 (72)	0	5 (426)	1	2 (251)	0	0 (37)	0
*asd-1(ok2299)*	6 (288)	16	0 (77)	0	5 (424)	0	3 (254)	0	1 (36)	0
*mec-8(u218)*	11 (293)	30	3 (82)	0	7 (422)	0	3 (252)	0	3 (37)	0
*mec-8(u303)*	30 (290)	43	8 (81)	0	24 (426)	1	7 (254)	0	3 (36)	0
*exc-7(rh252)*	15 (292)	17	6 (70)	0	3 (424)	0	5 (251)	0	3 (37)	0

^a^ Numbers in parenthesis indicate the number of events tested for that strain that had sufficient read count for analysis of splicing changes in that mutant strain relative to wild-type.

Overall, splicing is similar between wild-type animals and mutant animals (*fox-1(e2643)*, *asd-1(ok2299)*, *mec-8(u218)*, *mec-8(u303)*, and *exc-7(rh252)*)—less than 10% of annotated cassette exons showed any significant differences in exon inclusion in any mutant (Table 1). For example, in the *mec-8(u303)* mutant we identified 73 single cassette exons that show differential inclusion levels compared with wild-type worms. This suggests that these mutations do not greatly affect global splicing patterns and this is similar to what has been observed by other groups using a similar approach [48]. We also note that a third of the altered splicing events that we see in the mutants are splicing events that were found to change significantly across development [44], suggesting that these transcripts are affected by AS during normal development.

**Fig 2:**
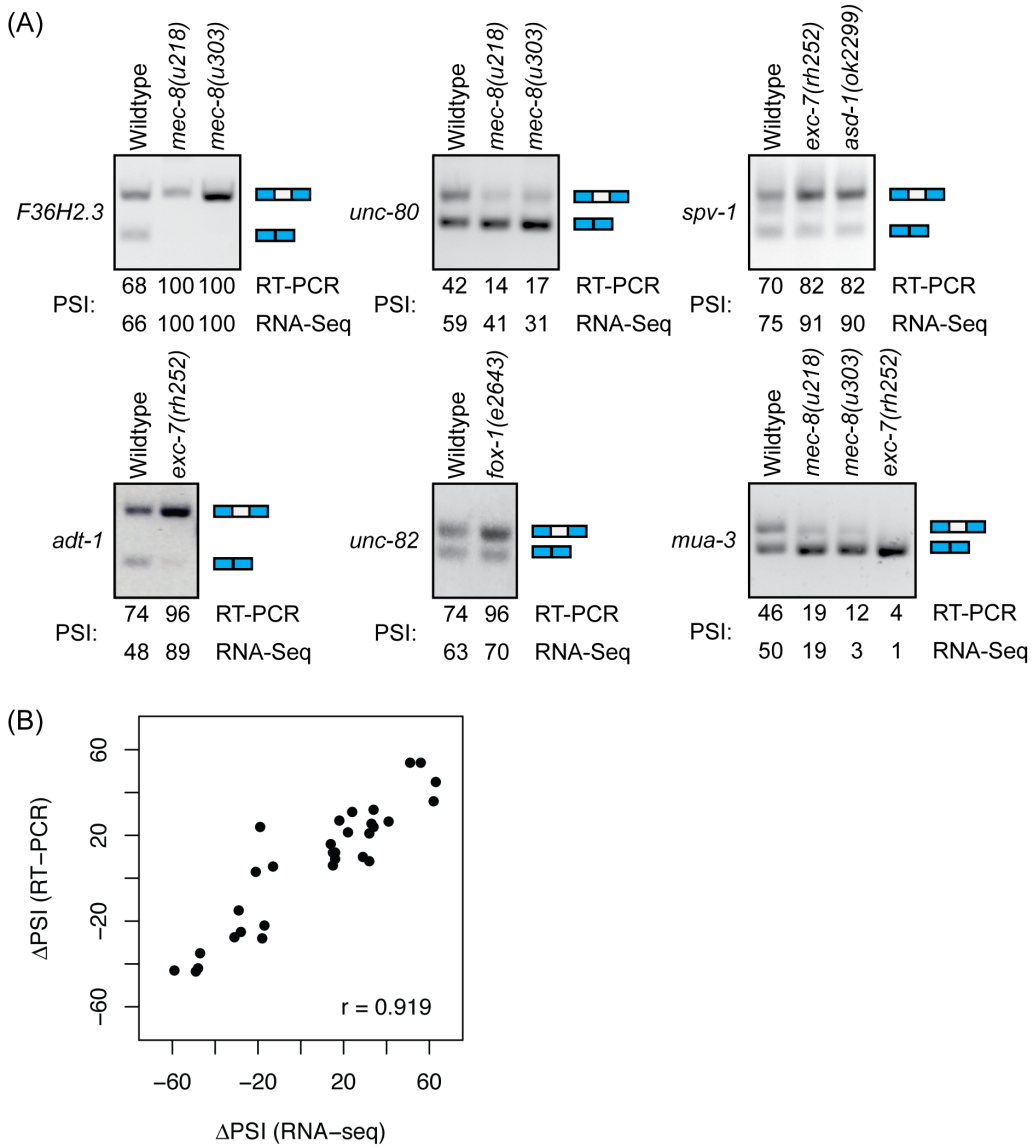
Validation of AS changes using semi-quantitative RT-PCR. (A) Semi-quantitative RT-PCR reproduces AS changes observed by RNA-seq. Independent RNA samples isolated from L4-sorted worms were used to validate RNA-seq data. (B) Changes in PSI values in wild-type and mutant worms show strong correlation with RNA-seq data. PSI values estimated from RT-PCR data was compared to RNA-seq derived PSI values and the strength of correlation was measured using Pearson’s correlation co-efficient.

**Fig 3:**
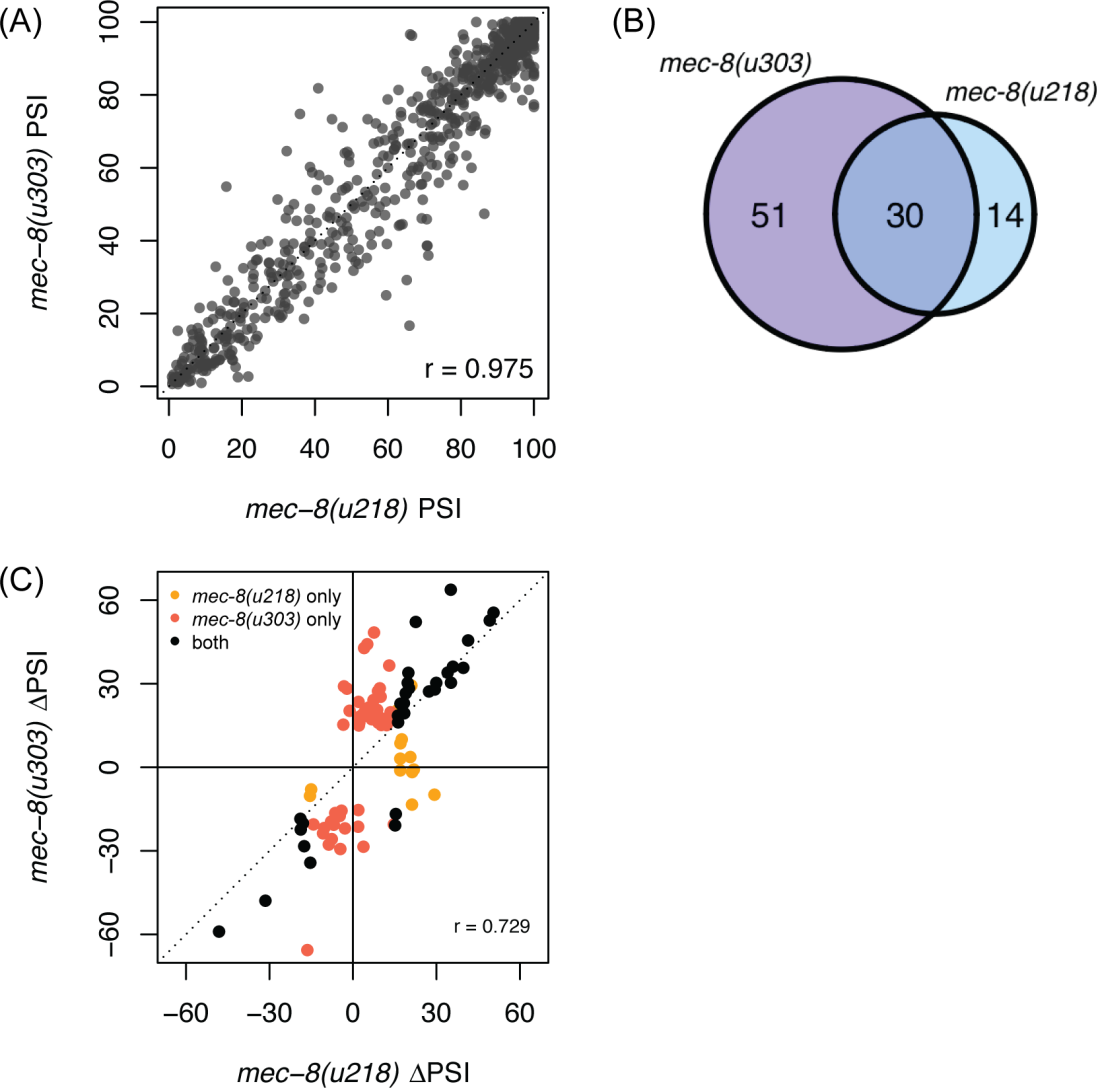
Overlap of AS changes in two different *mec-8* mutants. (A) PSI values of L4-staged AS events in both *mec-8* mutants are strongly correlated. The PSI values of all exons that have a PSI value of 1–99% in wild-type L4 worms were plotted and their PSI values in *mec-8(u218)* and *mec-8(u303)* mutant worms were compared using Pearson’s correlation. (B) Single and multiple exon skipping events overlap in both *mec-8* mutants. Illustrated are the number of exons that are differentially spliced in the two different *mec-8* mutants (p < 0.05, |ΔPSI| ≥ 15%) (C) Comparison of changes in PSI values for exons that are differentially spliced in one or both *mec-8* mutants. Black points denote AS events that are differentially spliced in both mutants, orange for events only differentially spliced in *mec-8(u218)*, and red for events only differentially spliced in *mec-8(u303)*. Pearson’s correlation was measured for all splice changes observed in either *mec-8* mutant.

We decided to focus on single and multiple cassette exon events, which comprise >70% of the AS changes identified in this study. To validate these AS changes that were identified in our RNA-seq data, we performed semi-quantitative RT-PCR on a subset of these events using independent RNA samples (Fig 2A). We were able to validate 80% (28/35) of the changes identified and found a strong correlation between percent-spliced-in (PSI) values obtained by the two methods (Fig 2B). Finally, to further validate our data, we also compared the PSI values of AS events between two different *mec-8* mutants that exhibit varying mutation severities. *mec-8(u218)* is a relatively weak allele whereas *mec-8(u303)* is a more severe loss-of-function allele [63]. The PSI values between the two sequenced samples are strongly correlated (r = 0.975) (Fig 3A), suggesting that the variability between samples should not greatly affect our conclusions. Importantly, there is a considerable overlap between the splice sites affected in the two *mec-8* mutants—as expected, the majority (30/44; 68%) of the changes found in the weaker *mec-8(u218)* strain reproduced in the more severe *mec-8(u303)* strain (Fig 3B) but more than half of the splice changes (51/81; 63%) were unique to the more severe strain. Moreover, of the AS changes detected in either strains, the changes in PSI values tended to be smaller in the *mec-8(u218)* mutant than in the *mec-8(u303)* mutant, consistent with differences in their mutation severities (Fig 3C).

So far, we have identified exons that are differentially spliced in the various SF mutants. However, these affected AS events could be due to either direct effects of the SF on these splicing targets or could instead be due to indirect, downstream effects, such as the aberrant splicing of other SFs or the mutant phenotype itself. We address this below.

### Enrichment of known binding motifs at spliced sites showing differential splicing in SF mutant strains

**Fig 4:**
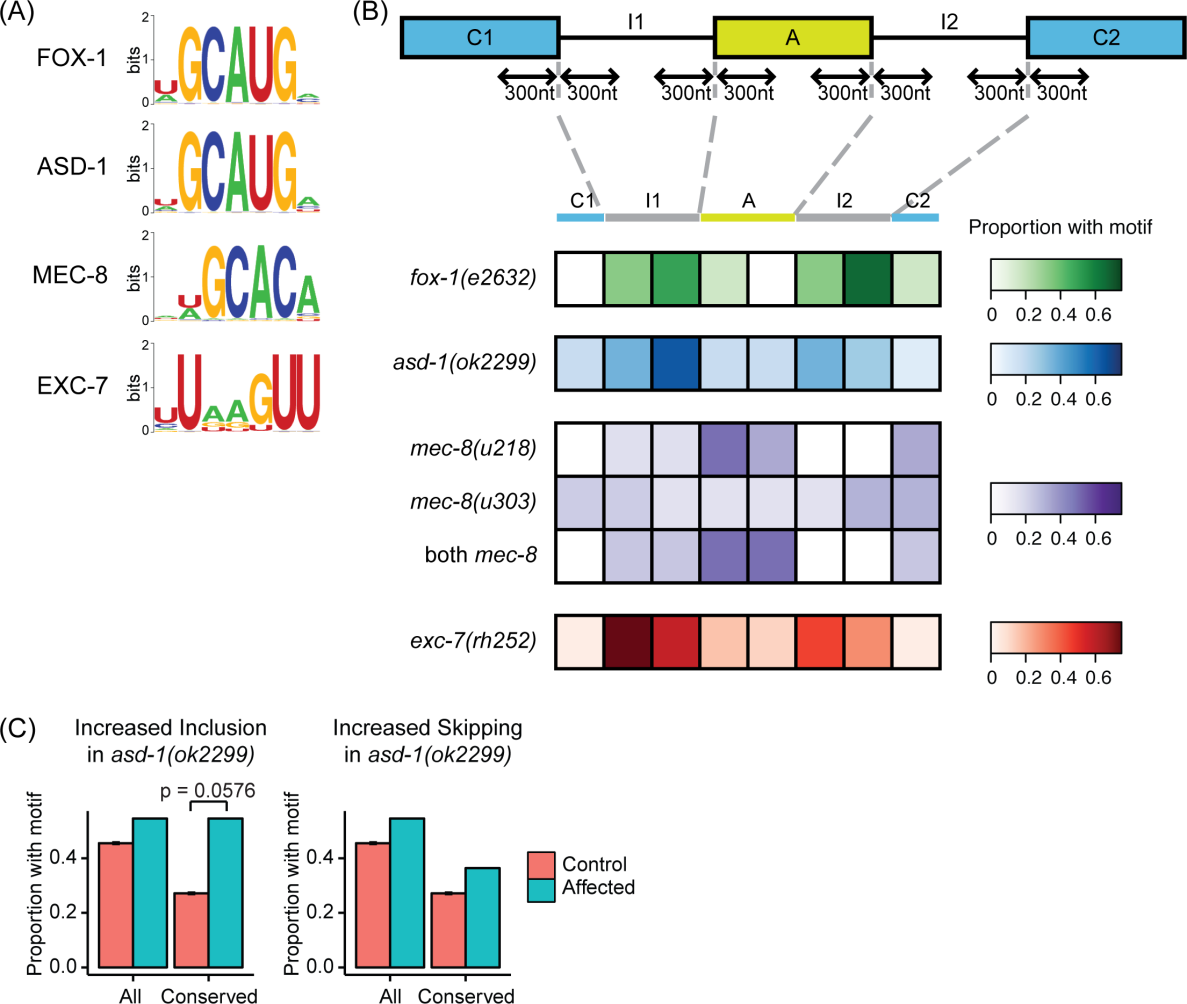
Binding motifs for SFs are enriched at splice sites surrounding differentially spliced exons. (A) RNAcompete-derived binding specificities of each SF. Motif logos were taken from the cisBP-RNA database [68]. (B) Positional bias for conserved binding motifs found at differentially spliced sites. Among the differentially spliced exons that have a conserved motif found at the alternative exon, flanking introns, or constitutive exons, the proportions of those AS events with one or more conserved motif(s) at that region are illustrated. The motif search was restricted to 300nt from the splice site and the *Caenorhabditis* species multiple alignment from the UCSC Genome Browser database [82] was used to identify conserved motifs, which we define here as the presence of the motif in 2 or more species other than *C*. *elegans*. (C) Conserved binding motifs are enriched in AS events that show increased inclusion in the *asd-1* mutant. This enrichment is not present if all instances of motifs (conserved and not conserved) are considered. In all comparisons, a random set of exons with PSI values matched with the affected exons was used as the control, and the data represented was the average taken from 100 randomized samples. Enrichment of AS events with motifs relative to control was tested using Fisher’s Exact Test.

SFs regulate splicing by binding at splicing regulatory elements in introns and exons to modulate selection of competing splice sites. We used this to distinguish between direct and indirect splicing regulation–we consider that any AS event that is affected by a SF but that has no consensus binding site is indirectly affected by that SF. We previously used the RNAcompete method [68,75] to define the *in vitro* binding specificities of our SFs of interest. We were able to obtain 7-mer binding motifs for each SF, reproducing both known [45,76,77] as well as novel consensus motifs (Fig 4A)—these data were previously published in a large survey of SF binding sites [68].We looked for the presence of these binding motifs at splice sites affected in the SF mutants, limiting our analysis to sequences 300nt proximal to the splice sites. Since these short sequence motifs are prevalent throughout the genome, we also used a phylogenetic approach to identify binding sites likely to be functionally relevant. We reasoned that just as functionally significant *cis-*acting transcriptional factor binding sites tend to be highly conserved across related *Caenorhabditis* species [78,79], functionally significant *cis-*acting SF binding sites would likewise be conserved. Similar approaches looking at sites conserved between *C*. *elegans* and *C*. *briggsae* have previously been used to identify motifs in *C*. *elegans* introns and exons that are important for splicing regulation [80,81]. Here, we used a multiple alignment of 5 *Caenorhabditis* species (*C*. *elegans*, *C*. *briggsae*, *C*. *remanei*, *C*. *sp*.*11* and *C*. *brenneri*) [82], to identify conserved sequences.

We first examined whether the set of splice events that are affected by mutation of any individual SF are enriched for the presence of its consensus binding motifs. There is a weak, but not significant, trend in this direction (Table 2) if we simply consider all possible recognition sequences in the genome—if we refine this to only examine the conserved (and thus likely functionally significant) motifs, for *exc-7* we find a significant enrichment of its binding motif at the affected splice sites relative to a control set of AS events (Table 2). For *mec-8(u303)*, we only observe a significant enrichment at splice sites where we see increased exon inclusion in the *mec-8* mutant (S3 Table). We note that while similar enrichments are seen for *asd-1* and *fox-1*, they are not significant. Overall, we find that only a minority of the exons with conserved SF binding motifs were differentially spliced in the respective SF mutants—this ranges from ~5 (10/189 for *asd-1*) to ~20% (9/43 for *mec-8*). We conclude that, just as found in other studies [48], many of the splice changes seen in any mutant strain are indirect consequences of loss of that SF rather than direct effects of that SF on the splicing of those transcripts.

**Table 2:** Proportion of affected AS events with SF binding motif at surrounding splice sites.

		With motif				With conserved motif			
Mutant strain	AS changes	Count	Prop.	Prop. in control^a^	*p*-value	Count	Prop.	Prop. in control^a^	*p*-value
*fox-1(e2643)*	17	10	0.588	0.465	0.236	6	0.353	0.294	0.339
*asd-1(ok2299)*	22	12	0.545	0.455	0.292	10	0.455	0.264	0.065
*mec-8(u218)*	44	20	0.455	0.364	0.185	4	0.0909	0.053	0.283
*mec-8(u303)*	81	34	0.420	0.383	0.337	9	0.111	0.056	0.099
*exc-7(rh252)*	38	31	0.816	0.789	0.463	23	0.605	0.377	0.012

^a^A control set of cassette exons with matched PSI values

We next examined the locations of the binding motifs for each of the 4 SFs and find positional biases for all 4 SFs (Fig 4B). For example, ASD-1 and FOX-1 binding motifs are enriched in the introns flanking cassette exons that show altered splicing in *asd-1* and *fox-1* mutants. In the case of *asd-1*, we observed enrichment of binding motifs specifically in introns that flank exons that show increased inclusion in the *asd-1* mutant (Fig 4C). We see a similar enrichment for EXC-7 motifs in introns that flank the cassette exons. MEC-8, however, shows a different pattern—MEC-8 sites appear to be enriched in the cassette exons themselves. In addition, for MEC-8, we observed more instances of increased exon inclusion in both *mec-8* mutants compared with increased skipping (S1 Fig), and the enrichment for MEC-8 motifs is specific for cases of increased exon inclusion (S3 Table). This suggests that MEC-8 primarily functions to repress exon inclusion, at least at the L4 larval stage, which is consistent with previous characterizations of MEC-8 as a repressor of exon inclusion [58–60].

We thus find that the splice events affected by mutation of each SF studied are enriched for the presence of conserved (and thus likely functionally relevant) binding motifs for the SFs. In addition, the location of the conserved binding motifs can shed light on the likely way in which each SF regulates splicing and where they appear to act on the pre-mRNA transcript. In the rest of this paper, we now focus on combinatorial regulation of splicing by the 4 SFs studied here.

### Multiple analyses suggest combinatorial interactions between SFs

In the sections above, we describe how we combined RNA-seq analysis of splicing patterns in strains carrying mutations in specific SFs with *in vitro* binding site data to identify the likely direct targets of several different *C*. *elegans* SFs. We next used these data to explore whether different SFs might regulate splicing of similar targets.

**Fig 5:**
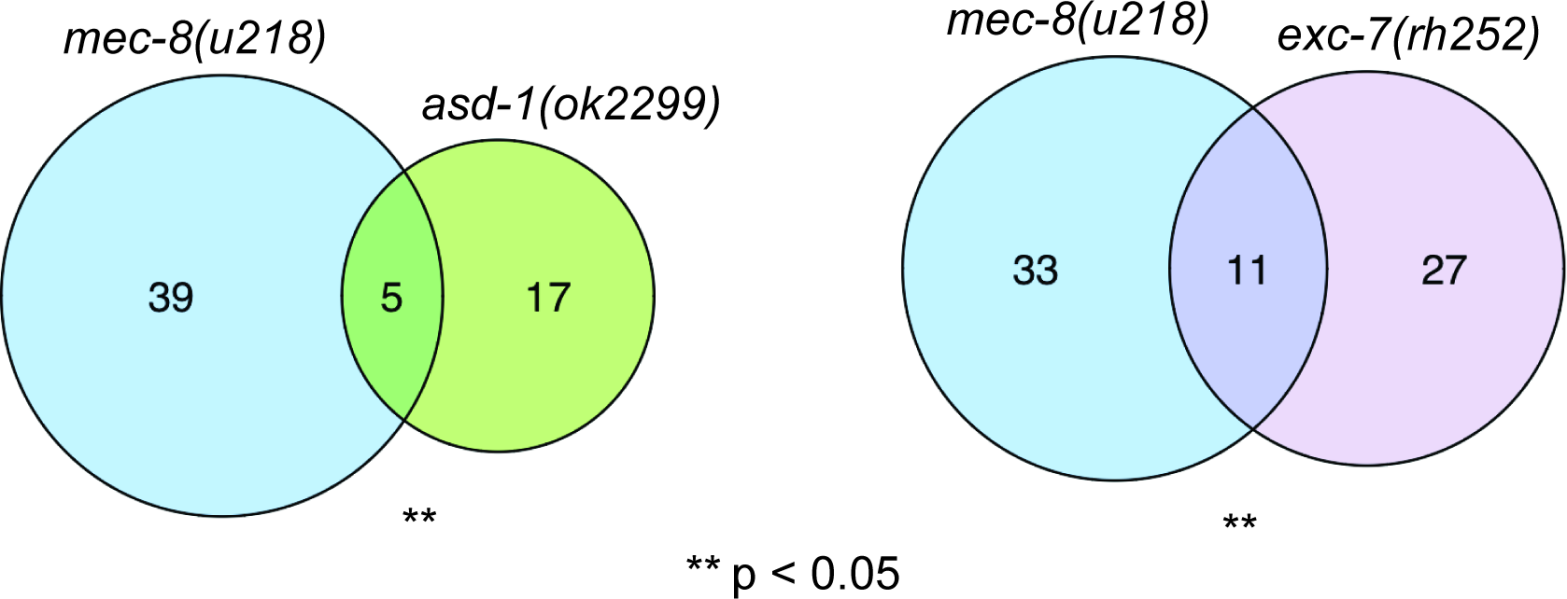
Enrichment of shared AS changes between SF mutants. The overlap of differentially spliced exons identified in *mec-8(u218)* and various other SF mutants was compared to that expected from a background of L4 AS events. A one-tailed hypergeometric test was used to calculate the significance of enrichment of overlapping exons.

We examined whether there were overlaps in the sets of splicing events affected in each of the mutant strains—if the same splice event is affected in more than one mutant, this might indicate that those SFs may combine to regulate that splice event. As expected, there was a significant overlap between splice sites affected in the two *mec-8* mutant strains. We also found a significant overlap between the splice events affected in either *asd-1* or *fox-1* mutants—this is unsurprising since they are highly related paralogs. Unexpectedly, however, we also observed a significant overlap between the splicing events affected in other pairs of splicing factors (Fig 5, S2 Fig). This suggests that these four SFs may regulate many of the same splicing events.

**Fig 6:**
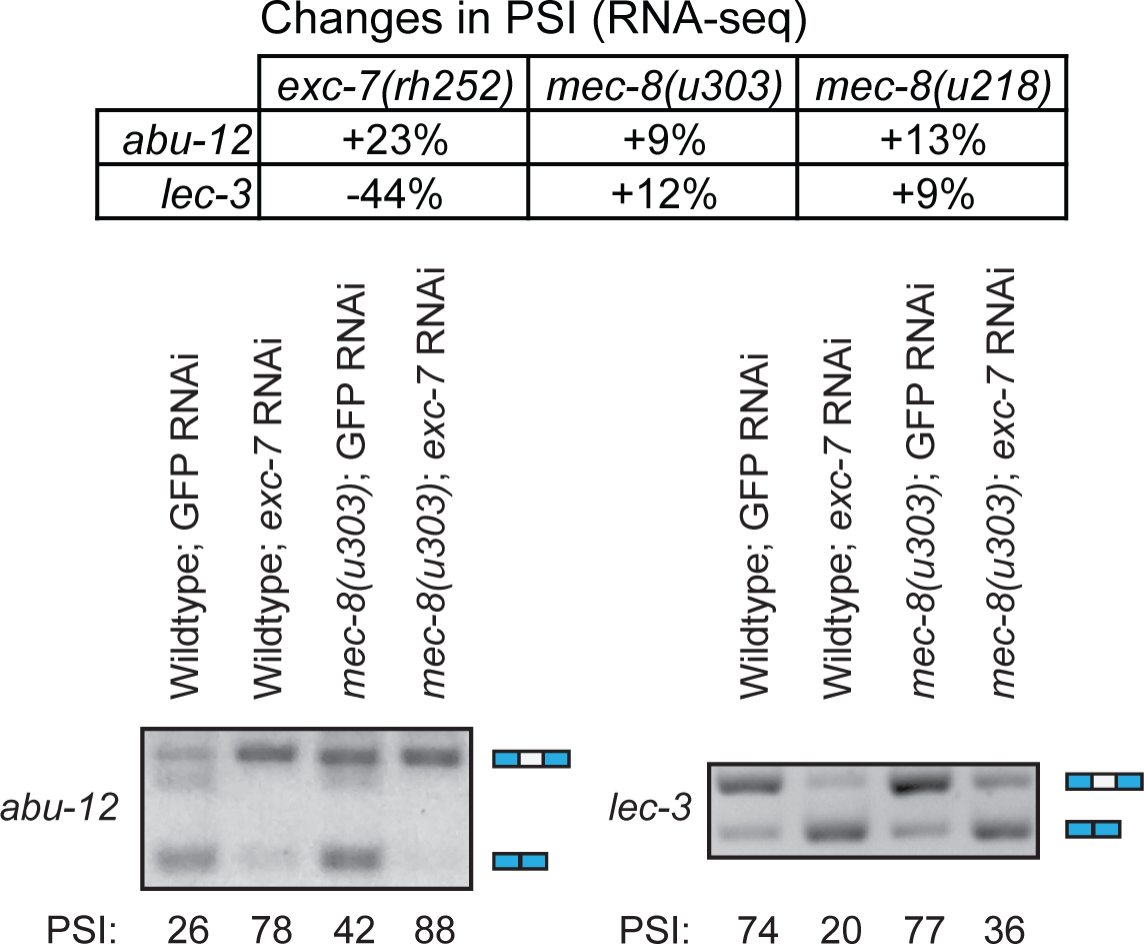
Loss of both EXC-7 and MEC-8 show an additive effect on shared targets. Synchronized L4 worms were subjected to RNAi on NGM plates seeded with dsRNA-expressing bacteria. GFP was used as a non-targeting control. The L4 progeny of these worms were then collected using a COPAS worm sorter, and their RNA isolated for use in these RT-PCR experiments.

To experimentally confirm this overlap in targets, we focused on MEC-8 and EXC-7. We identified individual splicing events that are affected by both the loss of EXC-7 and the loss of MEC-8 using our RNA-seq data and then used semi-quantitative RT-PCR to examine splicing at each individual splice site in strains lacking either EXC-7 or MEC-8 alone, or lacking both SFs at once. We identified several instances where loss of both splicing factors show an additive effect, suggesting distinct roles in regulating these AS targets (Fig 6). These include examples where both SFs either have the same effect on these targets, or differentially affect the target, with one resulting in increased exon inclusion and the other resulting in increased exon skipping. Our RNA-seq data show that SFs show much greater than expected overlaps in the splicing events that they affect and thus that multiple SFs often converge to regulate the same splicing events.

**Fig 7:**
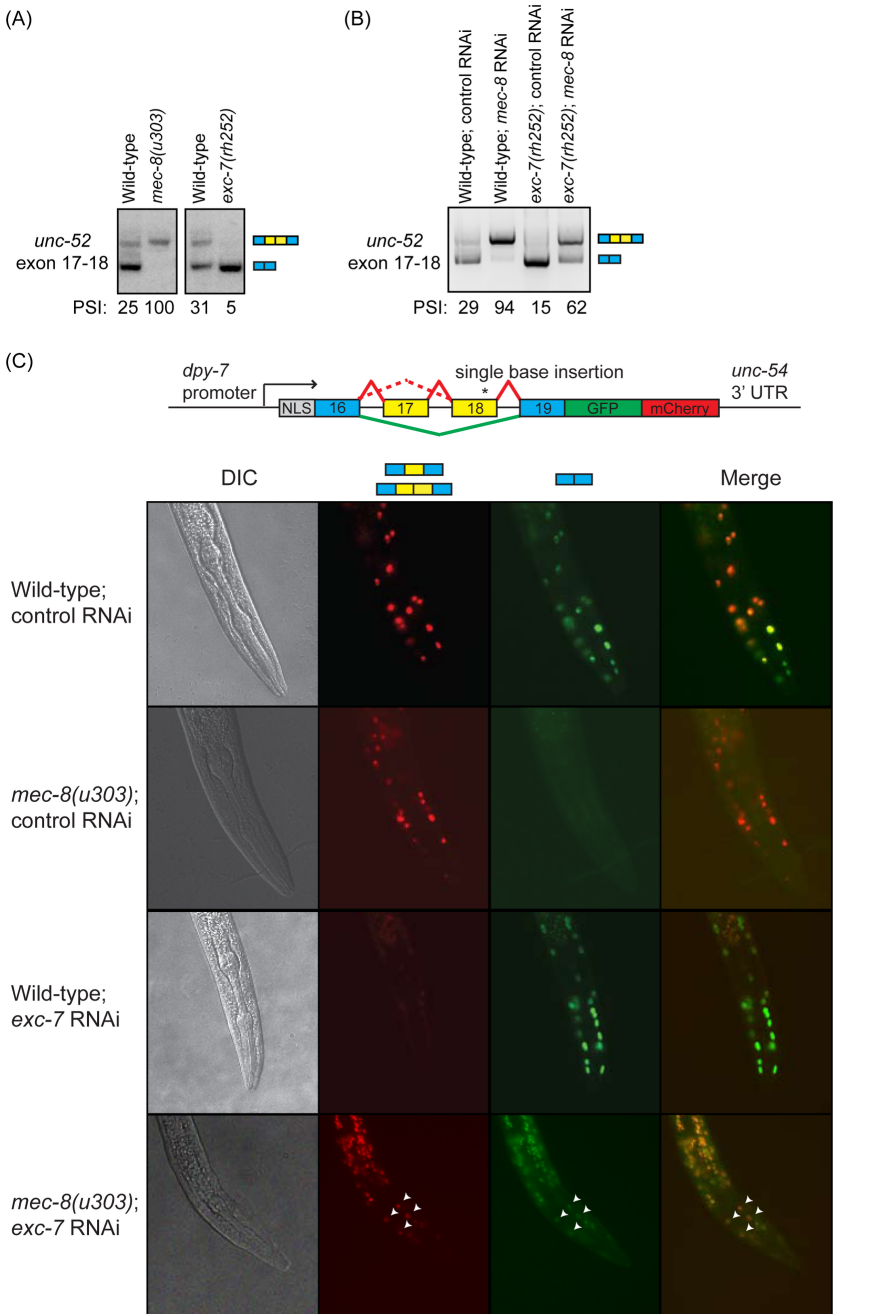
*mec-8* and *exc-7* combines to regulate splicing of *unc-52* exons. (A) *mec-8* and *exc-7* show antagonistic regulation of exons 17–18 in *unc-52* at the L1 stage. (B) Reduction of activity of both *mec-8* and *exc-7* show additive effects on *unc-52* splicing patterns at the L1 stage. (C) Study of *unc-52* splicing patterns *in vivo* using a bichromatic splicing reporter. The bichromatic splicing reporter was constructed using the same method as described by Norris *et al*. [48]. Skipping of exons leads to expression of GFP, while inclusion of exon(s) results in a frameshift that leads to readthrough of the GFP reading frame, and resulting expression of mCherry. Worms were subjected to RNAi at the L4 stage, and late L1/L2 worms were imaged. A dsRNA that targets a *C*. *briggsae* gene was used as a non-targeting control. Transgenic *mec-8* mutant worms show increased inclusion of the *unc-52* minigene exons in hypodermal cells, while worms treated with *exc-7* RNAi show increased exon skipping. Transgenic *mec-8* worms treated with *exc-7* RNAi show both exon-included and exon-skipped isoforms in hypodermal cells. Examples of hypodermal cells that express both isoforms are highlighted with white arrowheads.

One caveat of these analyses is that both RNA-seq and RT-PCR approaches examine splicing at the level of the whole animal. For example, we find that loss of *mec-8* results in increased inclusion of exons 17 and 18 of *unc-52* (a well known result [58,59]), loss of *exc-7* results in increased skipping of that exon (Fig 7A), and loss of both *mec-8* and *exc-7* together results in partial skipping and partial inclusion (Fig 7B), a so-called “antagonistic interaction” [48]. This partial inclusion in the double mutant could either be the result of some cells having complete skipping while others show complete inclusion or many cells could each be expressing a mixture of the isoforms. To examine this, we made a bichromatic splicing reporter construct that allows us to assess the level of *unc-52* exon 17–18 inclusion or skipping *in vivo* in each cell. We find that in wild-type animals, hypodermal cells typically express either one or other isoform and that there is a mix of ‘inclusion’ cells and ‘skipped’ cells (Fig 7C). As expected [58,59], when *mec-8* is mutated, there is a major shift towards exon inclusion—we cannot detect any exon-skipped isoforms but only see the exon-included form (seen as red signal in Fig 7C). When we target *exc-7* by RNAi we see the reverse—cells mostly express the exon-skipped form (seen as green signal in Fig 7C). However, when we knock down *exc-7* by RNAi in a *mec-8* mutant, in addition to cells that appear to express either entirely one or other isoform, we also see cells that express a mixture of isoforms suggesting that MEC-8 and EXC-7 can combine to affect splice site choice in a single cell.

To explore the overlap in targets between the 4 diverse SFs in our study in greater detail, we examined whether the binding motifs for each of the 4 SFs co-occur more than randomly expected—we find this to be the case (Table 3). Since there are high quality binding motifs for more SFs in *C*. *elegans* than the 4 studied in depth here, we expanded our analysis to include all known or predicted *C*. *elegans* SFs that had high quality *in vitro* binding motifs that were defined using RNAcompete or inferred based on sequence similarity to RBPs with defined binding motifs [68]—these 16 SFs are listed in S4 Table and span a variety of biological functions. We find that many predicted SF binding sites co-occur more than randomly expected (see S5 Table) suggesting that there could be extensive co-regulation of splicing targets by SFs. To gain some insight into how such co-regulation might occur, we examined both the distance between co-occurring motifs and their relative orientation.

**Table 3:** SF binding motifs significantly co-occur at sites surrounding cassette exons.

Filter	SF Motif 1	SF Motif 2	Expected^a^	Actual	*p*-value^b^
All motifs	FOX-1/ASD-1	MEC-8	103	117	0.0145
Conserved motifs	FOX-1/ASD-1	MEC-8	10	21	0.000151
All motifs	FOX-1/ASD-1	EXC-7	230	246	0.000673
Conserved motifs	FOX-1/ASD-1	EXC-7	59	96	2.37E-11
All motifs	MEC-8	EXC-7	190	201	0.0129
Conserved motifs	MEC-8	EXC-7	13	21	0.00572

^a^ Expected co-occurrence of 2 motifs was calculated using a multiplicative model

^b^ A cumulative hypergeometric function was used to calculate significance of motif co-occurrence

**Fig 8:**
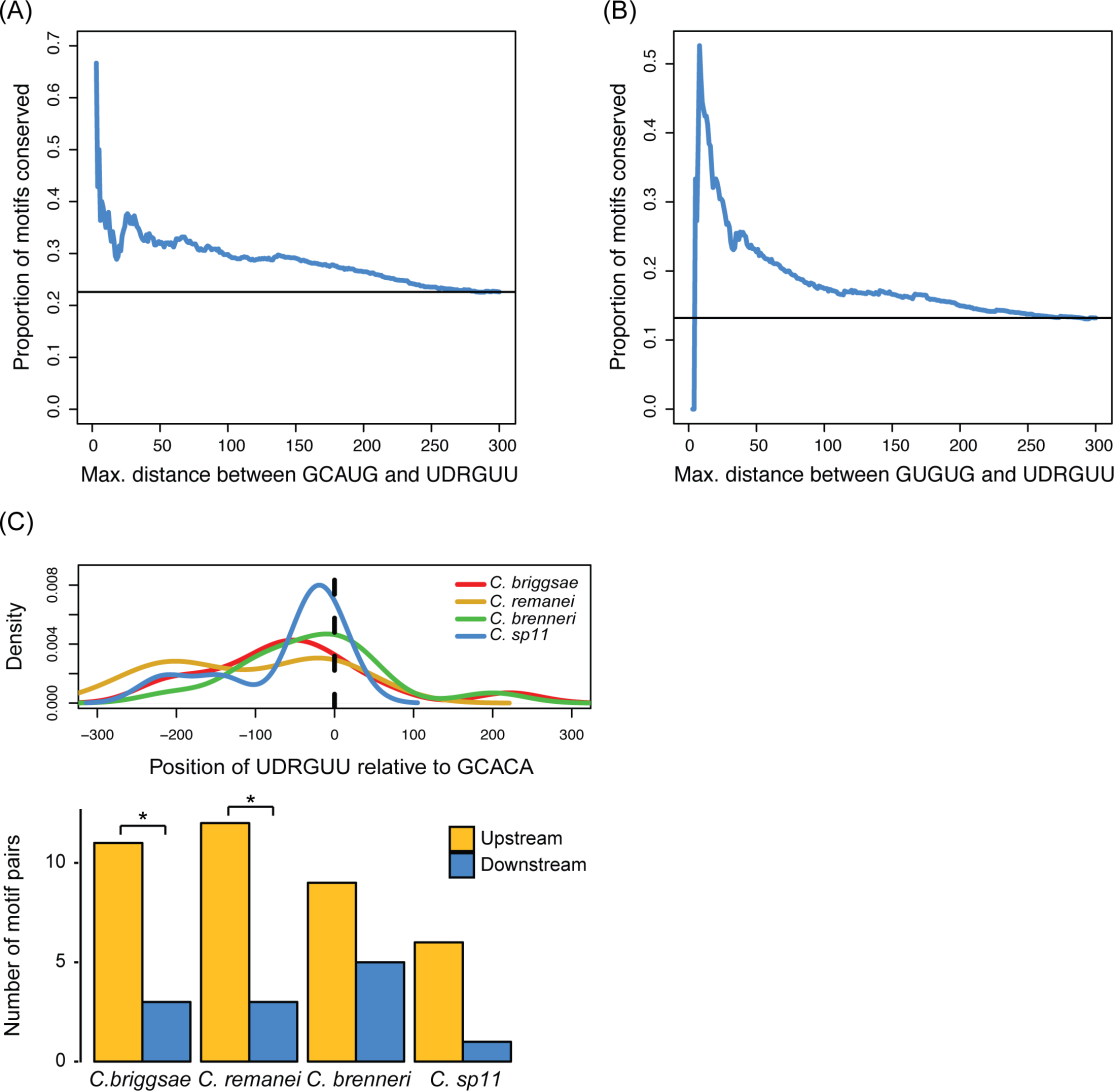
The distribution patterns of binding motifs for co-occurring SF motifs vary among different SF pairs. (A-B) Binding motifs for EXC-7 and SUP-12, and for FOX-1/ASD-1 and EXC-7 were more likely to be conserved across *Caenorhabditis* species if they were found closer together. The proportions of motif pairs where both motifs were conserved across species were plotted cumulatively, where pairs of motifs with inter-motif distances less than or equal to the x-axis values were included at each point. The black horizontal line represents the proportion of conserved motifs among all considered motif pairs. (C) While the distances between EXC-7 and MEC-8 recognition motifs fall within a wide range, the relative position of the EXC-7 motif as being upstream of the MEC-8 motif is conserved in other *Caenorhabditis* species. For each AS event with co-occurring EXC-7 and MEC-8 binding motifs, the position of a MEC-8 recognition motif was plotted at 0 on the x-axis of the density plot and the position of the co-occurring EXC-7 motif was plotted relative to that. A positive value indicates positioning of an EXC-7 motif downstream of a MEC-8 motif, while a negative value indicates positioning of an EXC-7 motif upstream of a MEC-8 motif. Significance was calculated using a binomial test (* = p < 0.05).

We first analyzed the average distance between co-occurring motifs—if motifs of two SFs tend to co-occur in close proximity, it suggests that they could cooperate to regulate splicing. Moreover, as MEC-8 and EXC-7, and ASD-1 and EXC-7, were found to physically interact [83], close proximities of binding sites for these SFs could indicate cooperative binding. We found the majority of EXC-7 and SUP-12, and FOX-1/ASD-1 and EXC-7 binding motifs in introns flanking cassette exons to co-occur within 60nt of each other (S3 Fig). More importantly, we also find that the closer their binding sites are together, the more likely the binding sites are to be conserved across multiple species at the sequence level (Fig 8A and 8B). This suggests that the proximity of the sites is likely to be key for their functions and for the way in which the SFs regulate these splicing events.

We next examined the relative positions of pairs of SF motifs—for example, if binding motifs co-occur, do they tend to show any bias in their relative order, and is this ordering conserved? We find that this is indeed the case for certain combinations of SF motifs (Table 4). For example, when an EXC-7 motif is upstream of a MEC-8 motif in the same intron, the EXC-7 motif also tends to be found upstream of the MEC-8 motif in other species even if the spacing between the motifs varies (Fig 8C). In some cases this ordering of SF sites appears to be unidirectional: for example the EXC-7 site always tends to be upstream of the MEC-8 site. In other cases, while the ordering is conserved for any individual pre-mRNA, the ordering varies between pre-mRNAs—for example, in cases where a GCAUG (FOX-1/ASD-1) motif is 5’ to a CUAAC (ASD-2) motif in *C*. *elegans* it will tend to be 5’ to a ASD-2 motif in other species, and where a FOX-1/ASD-1 motif is 3’ to an ASD-2 motif in *C*. *elegans*, it tends to be 3’ to a ASD-2 motif in other species. Our data thus suggest that SFs may combine to affect the same target splicing event in two distinct ways: either by binding closely located sites (such as for EXC7 and SUP-12, or FOX-1 and EXC-7) or by binding completely distinct regions of the pre-mRNA, often in some ordered manner (like EXC-7 and MEC-8).

**Table 4:** SF motifs with conserved ordering in introns flanking alternative exons.

Bidirectional	
12/78^a^^,^^b^	
SF Motif 1	SF Motif 2
ETR-1	FOX-1/ASD-1
ETR-1	SUP-12
ETR-1	ASD-2
EXC-7	FOX-1/ASD-1
EXC-7	SUP-12
EXC-7	SAP-49
FOX-1/ASD-1	ASD-2
SUP-12	FOX-1/ASD-1
SUP-12	SUP-26
SUP-12	ASD-2
SUP-12	MEC-8
TIAR-1/2/3	SUP-12
Unidirectional	
33/78^a^^,^^b^	
SF Motif 1	SF Motif 2
ASD-2	SUP-26
ASD-2	MEC-8
ETR-1	SUP-26
ETR-1	TAG-262
ETR-1	SAP-49
ETR-1	MEC-8
EXC-7	ETR-1
EXC-7	SUP-26
EXC-7	TAG-262
EXC-7	ASD-2
EXC-7	MEC-8
FOX-1/ASD-1	SUP-26
FOX-1/ASD-1	TAG-262
FOX-1/ASD-1	MEC-8
MEC-8	MSI-1
RSP-3	ASD-2
SUP-12	TAG-262
TIAR-1/2/3	EXC-7
TIAR-1/2/3	ETR-1
TIAR-1/2/3	FOX-1/ASD-1
TIAR-1/2/3	TAG-262
TIAR-1/2/3	ASD-2
TIAR-1/2/3	SAP-49
TIAR-1/2/3	MEC-8
TIAR-1/2/3	UNC-75
UNC-75	EXC-7
UNC-75	ETR-1
UNC-75	FOX-1/ASD-1
UNC-75	SUP-12
UNC-75	ASD-2
UNC-75	SAP-49
UNC-75	MEC-8
UNC-75	MSI-1

^a^ p<0.05, Chi-squared Test

^b^78 refers to the total number of pairwise comparisons done to test for presence of conserved ordering among 13 unique motifs.

**Fig 9:**
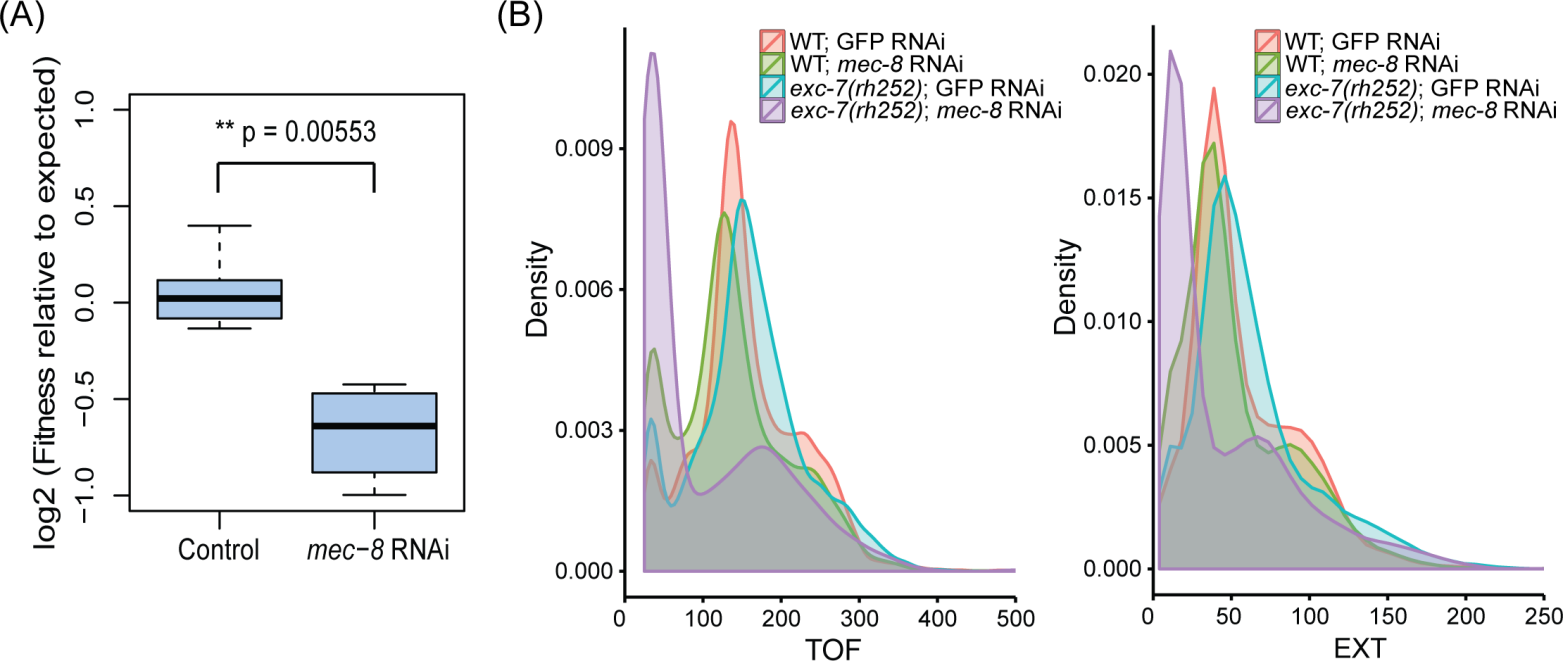
*exc-7* and *mec-8* genetically interact in a fitness assay. An average of 10 L1 worms were grown in bacteria expressing dsRNA for 5 days with GFP used as a non-targeting control. The resulting population sizes were counted using a COPAS worm sorter, and values were normalized to the GFP control and used as a proxy for fitness as previously described [102,103]. (A) A multiplicative model was used to calculate the expected fitness value for each mutant/RNAi condition, and *exc-7* mutant worms subjected to *mec-8* RNAi exhibit a more severe fitness defect than expected (student’s t-test). A log fitness score of 0 implies no interaction, and a negative log score implies a negative interaction. (B) Time-of-flight (TOF) and extinction (EXT) values of worm populations were also measured using the worm sorter. Lower TOF and EXT values for *exc-7* mutant worms under *mec-8* RNAi condition suggest a growth defect more severe than that observed in worms with loss of either *exc-7* or *mec-8* alone.

To partly test the possible functional significance of any combinatorial interaction of SFs with their pre-mRNAs, we examined the effects of reducing the activity of either single SFs or combinations of SFs on the phenotype of *C*. *elegans* using a combination of genetic mutations and RNAi. We tested 48 pairs of SFs in this way and identified 6 clear functional interactions (Table 5, S6 Table). For example, EXC-7 and MEC-8 have overlaps in the splice events that they affect and in the transcripts that contain their binding sites. We find that reducing activity of either SF alone only has a weak effect on the fitness of the animals; however, reduction in activity of both together results in a much more severe fitness defect than expected with smaller brood sizes (Fig 9A) and growth defects (Fig 9B). This clear genetic interaction between these two SFs suggests that they functionally cooperate. We note that the molecular basis for this functional cooperation is not clear from these results—it might be that they co-regulate the same targets, and thus the double reduction has more severe consequences on a shared set of targets, or that the observed synergy is simply due to increased numbers of transcripts with altered splicing thus that the overlaps in their splice targets may indeed be functionally relevant. We find 5 other such genetic interactions (*asd-1* and *asd-2*, *asd-1* and *etr-1*, *asd-1* and *rsp-3*, *mec-8* and *rsp-3*; *mec-8* and *asd-2*; Table 5). We note that the genetic interactions that we observe all occur between SFs that bind to distinct regions of the pre-mRNAs and that we observed no such interactions between SFs whose sites appear to be closely located on pre-mRNAs consistent with close cooperation.

**Table 5:** Genetic interactions identified in fitness screen. An interaction score of 0 implies a maximal negative interaction and a score of 1 implies no genetic interaction.

Mutant	RNAi	Interaction Score
*asd-1*	*asd-2*	0.163
*asd-1*	*etr-1*	0.342
*asd-1*	*rsp-3*	0.345
*mec-8*	*rsp-3*	0.325
*mec-8*	*asd-2*	0.402
*exc-7*	*mec-8*	0.623

In summary, we find multiple lines of evidence that indicate that many AS events are regulated by multiple SFs during *C*. *elegans* development. There is a significant overlap in the sets of splice events affected by mutation of each SF suggesting that they may regulate the same AS events. There is also clear enrichment for co-occurrence of the defined binding motifs of different SFs in the same transcripts. In addition (see analysis in next section below), the genes that contain many SF motifs in their introns are much more likely to show significant changes in their AS patterns across development [44]. Finally, we find that SFs may co-regulate AS events in two distinct ways. Some SFs have binding motifs that co-occur within close proximity in the same intron—this includes FOX-1/ASD-1 and EXC-7, EXC-7 and SUP-12 (S3 Fig) for example. The closer their sites, the more likely they are to be conserved and thus functionally relevant (Fig 8A and 8B). However, other SFs like EXC-7 and MEC-8 appear to functionally interact in a very different way—their binding sites often co-occur, but the distance between them is often quite long and the distance is not conserved. What is conserved is the orientation of their sites—EXC-7 sites tend to be upstream of MEC-8 sites when they occur in the same transcript (S4 Fig), and this orientation is maintained across long evolutionary distances. Taken together, these data all point to many transcripts having complex regulation of their splice patterns—we next examined whether this complex regulation is in any way restricted to particular classes of genes.

### Enrichment of specific gene classes among targets with co-occurring motifs

**Fig 10:**
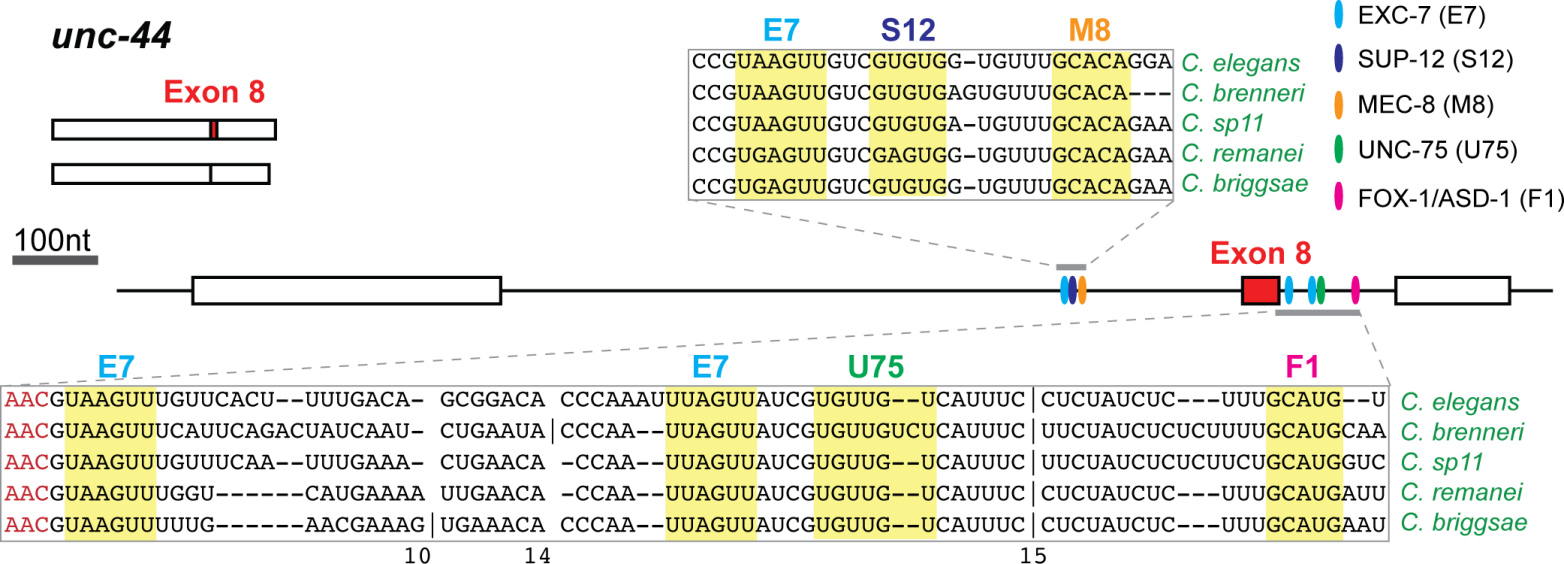
Binding motifs for multiple SFs co-occur at introns flanking alternative exons. Example of co-occurring motifs that are conserved at introns flanking alternatively spliced exons. Sequence alignments were taken from the UCSC Genome Browser. A vertical line indicates added bases not shown, with the number of bases indicated below. Each oval represents a conserved binding motif, and only motifs that are present in introns and within 300nt of each splice site are illustrated. The alternative exon was expanded for illustrative purposes and is not drawn to scale.

In the above analyses, we identified AS events with conserved consensus binding motifs for multiple SFs that suggest potential splicing co-regulation by these SFs. We noticed that many of these potentially co-regulated cassette exons are found in functionally coherent groups of genes that appear to be highly enriched in genes involved in proper neuromuscular or cytoskeletal functions (S7 Table). For instance, many of these genes are required for proper locomotion in the animal; mutations in many of these genes lead to an uncoordinated (*unc*) phenotype, such as *unc-2* and *unc-44*, which have conserved binding motifs for FOX-1/ASD-1, MEC-8, EXC-7, SUP-12 and UNC-75 (Fig 10, S5 Fig).

To examine this further, we used Gene Ontology (GO) [84] to identify enriched functional categories among these potentially co-regulated targets. We found the set of genes with co-occurring motifs to be enriched for several classes of genes (Table 6, S8 Table). This includes genes involved in cytoskeletal structures that are key for neuron growth and function (e.g. *unc-44*, *nab-1*) [85–87] and genes that are key ion channels in the neuromuscular system (e.g. *unc-2*, *unc-49*, *tmc-1*, *exp-2*) [88–91], suggesting that neuromuscular genes tend to have more complex AS regulation. While some genes with key roles in the neuromuscular are known to have long and complex transcripts (e.g. *ttn-1* has a 55kb coding sequence and 66 coding exons [55,92], *unc-22* has a 20kb coding sequence and 34 exons [55]), this is not the reason for the observed enrichments: genes in these specific functional classes are more likely to contain motifs for multiple SFs than a random set of genes with similar total intron lengths (Table 7). For example, 36% of genes with the cytoskeletal protein binding GO term have both a conserved ASD-1/FOX-1 and EXC-7 motif, while only 8% of the intron length-matched set of genes without that GO term have the two motifs co-occurring, suggesting that the GO enrichment results were not merely due to those genes having larger motif search spaces and is instead likely biologically relevant.

**Table 6:** Enrichment of GO terms among genes with co-occurring SF binding motifs at sites surrounding alternative exons.

**ASD-1/FOX-1 and EXC-7 motifs**				
GO MF ID	p-value	Count^a^	Size^b^	Term
GO:0008092	4.16795E-05	10	28	Cytoskeletal protein binding
GO:0008017	0.010174876	3	6	Microtubule binding
GO:0051015	0.016717372	3	7	Actin filament binding
GO:0015085	0.019924034	4	13	Calcium ion transmembrane transporter activity
GO:0022836	0.021630321	8	42	Gated channel activity
GO:0022891	0.021845654	13	85	Substrate-specific transmembrane transporter activity
GO:0005216	0.035243899	10	63	Ion channel activity
GO:0005215	0.036937705	15	110	Transporter activity
GO:0022890	0.040659259	8	47	Inorganic cation transmembrane transporter activity
GO:0022803	0.047125052	10	66	Passive transmembrane transporter activity
**MEC-8 and EXC-7 motifs**				
GO MF ID	p-value	Count^a^	Size^b^	Term
GO:0015085	0.020514712	2	13	Calcium ion transmembrane transporter activity
GO:0022832	0.034367753	2	17	Voltage-gated channel activity
GO:0022890	0.045311572	3	47	Inorganic cation transmembrane transporter activity

^a^ Number of genes with GO term in set of genes with both motifs

^b^ Total number of genes with GO term in the background set used for comparison.

**Table 7:** Genes with specific GO terms are enriched for co-occurring motifs.

**ASD-1/FOX-1 and EXC-7 motifs**		
Term	Proportion with conserved motifs	
	With GO	Without GO^a^
Cytoskeletal protein binding	0.357 (10/28)	0.0760
Microtubule binding	0.500 (3/6)	0.0647
Actin filament binding	0.429 (3/7)	0.0737
Calcium ion transmembrane transporter activity	0.308 (4/13)	0.156
Gated channel activity	0.190 (8/42)	0.0717
Substrate-specific transmembrane transporter activity	0.153 (13/85)	0.0811
Ion channel activity	0.159 (10/63)	0.0824
Transporter activity	0.136 (15/110)	0.0913
Inorganic cation transmembrane transporter activity	0.170 (8/47)	0.0995
Passive transmembrane transporter activity	0.152 (10/66)	0.0835
**MEC-8 and EXC-7 motifs**		
Term	Proportion with conserved motifs	
	With GO	Without GO^a^
Calcium ion transmembrane transporter activity	0.154 (2/13)	0.00346
Voltage-gated channel activity	0.118 (2/17)	0.0203
Inorganic cation transmembrane transporter activity	0.0638 (3/47)	0.0101

^a^ As control, an equal number of AS genes with a similar intron length distribution as those of AS genes with the specific GO term was randomly selected from the set of genes without the GO term. Proportions indicated were taken from 1000 random samples.

**Fig 11:**
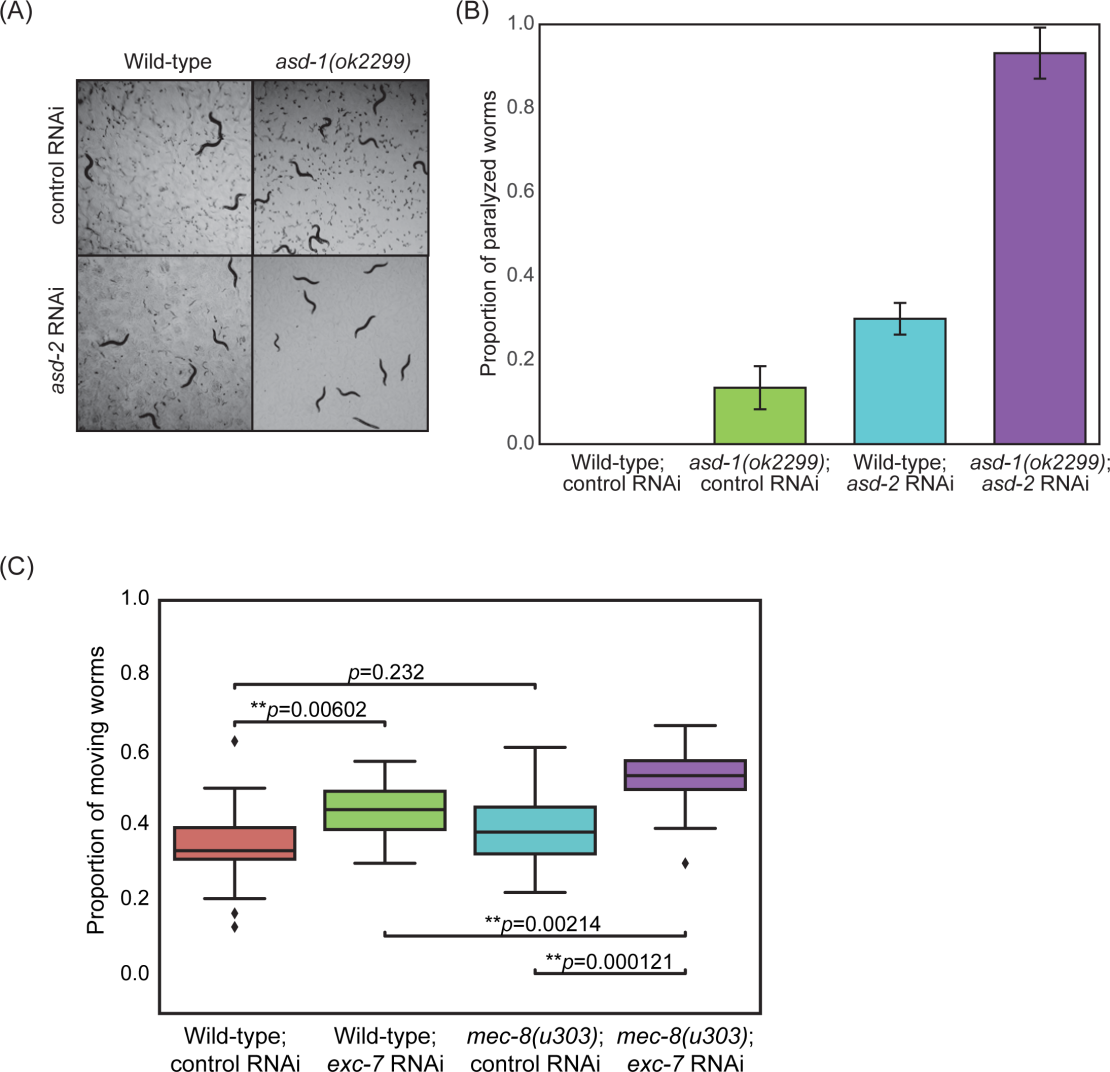
Combinatorial effects on movement and function of the neuromuscular system. (A) Loss of *asd-1* and *asd-2* results in sterile animals. Wild-type and mutant L1 worms were subjected to RNAi on NGM plates seeded with dsRNA-expressing bacteria for 4 days. GFP was used as a non-targeting control. (B) Reduction in activity of both *asd-1* and *asd-2* results in an increased number of paralysed worms. As in (A), L1 worms were subjected to RNAi on NGM plates and phenotypes of these worms were scored 3 days past adulthood. Worms were considered to be paralysed if the body of the worm was non-responsive to prodding with a worm pick. (C) Loss of *mec-8* and *exc-7* result in increased defects in synaptic transmission. Wild-type and mutant L4 worms were subjected to RNAi on NGM plates, and after 2 days, their L1 progeny were used for the drug assay. ~100 L1 worms were treated with 2mM aldicarb in a liquid assay for 3h. After 3h, their movements were scored depending on whether they exhibited wild-type-like movement similar to that observed in the no drug (DMSO only) control. The proportion of worms in each well that exhibited normal movement were plotted, with a total of 20 wells scored across 4 biological replicates. A student’s t-test was used to compare differences in proportions.

These data suggest that the 4 SFs we studied tend to interact frequently to affect splicing of genes with roles in the neuromuscular system. To further explore this, we examined whether some of the splice factors that show genetic interactions have combinatorial effects on neuromuscular system function rather than simply on the broad phenotypes of growth rate or viability as we had done above. We find that when either *asd-1* or *asd-2* activity is lost alone (either through mutation or RNAi), the worms have near-normal movement. However reduction in activity of both *asd-1* and *asd-2* results in sterile adult worms (Fig 11A) that are almost completely paralysed (quantified in Fig 11B) indicating that these two factors do indeed show a strongly synergistic effect on movement. In the case of *mec-8* and *exc-7*, we examined how these factors affect a specific aspect of neuromuscular system function, cholinergic transmission. Acetylcholine (ACh) is the major neurotransmitter at neuromuscular junctions (NMJs)—drugs such as aldicarb that alter ACh levels at NMJs result in paralysis [93,94]. Previous studies showed that *exc-7* mutants show reduced sensitivity to aldicarb [48,66]–we decided to test whether *mec-8* and *exc-7*, two factors that share many targets and whose binding sites co-occur frequently and that also show genetic interactions in our hands (see Fig 9) might have combinatorial effects on aldicarb sensitivity. We find this is indeed the case (Fig 11C)—reduction of activity of either *mec-8* or *exc-7* causes a small decrease in aldicarb sensitivity but reduction of activity of both results in a more severe reduction. We conclude that at least in these two cases, there is evidence that these factors combine to affect phenotypes that are relevant to neuromuscular system function.

Together, all these data combine to suggest that there is complex splicing regulation of genes involved in the neuromuscular system and in the cytoskeleton, whereas most other splice events in other sets of genes seem to be primarily regulated by just one of these SFs. This finding has a clear caveat however: the 4 SFs in this study all have characterized functions in neuromuscular development and this could explain the prevalence of potential shared targets with similar functions. For example, since MEC-8 and EXC-7 have key roles in proper neuronal function (in mechano- and chemo-sensation [62,64] and in synaptic transmission [66] respectively) it may not be particularly surprising that their splicing regulatory pathways may converge upon genes encoding ion channels, many of which are involved in neurotransmission. We therefore cast our net further and examined the set of all 16 SFs that had high quality *in vitro* defined or inferred binding motifs.

We first identified the sets of genes that had conserved intronic binding motifs for multiple of the 16 SFs. We find that these genes often show complex splicing regulation across development—for example ~40% of the genes with 4 or more distinct SFs motifs (43/108) showed significant changes to their alternative splicing patterns across development in a previous study [44]. This is a highly significant enrichment (p = 4.85×10^−22^, one-sided hypergeometric test) compared to 12.9% of background AS genes showing developmental regulation. This shows that the number of SFs that we predict to be able to bind and thus regulate any transcript correlates well with the complexity of the splicing changes for that transcript across development. We next identified GO terms that were enriched in these genes that have multiple SF motifs at introns flanking alternative exons (all results in S9 Table). Intriguingly, we found similar results to our initial analysis that only examined 4 SFs—many of the genes that are predicted to be targets of multiple SFs have GO terms consistent with a role in the neuromuscular system and regulation of the cytoskeleton. These include neurogenesis (GO:0022008), axon fasciculation (GO:0007413), regulation of locomotion (GO:0040012), cytoskeletal protein binding (GO:0008092) and transmembrane transporter activity (GO:0022857), and include many genes with well-characterized roles in neuronal functions such as *unc-44*, *unc-2*, and *fust-1* (see Fig 10, S5 Fig). We thus suggest that, at least in *C*. *elegans*, genes with key roles in the neuromuscular system appear to have highly complex regulation of splicing. We note that the set of genes whose splicing was previously reported to change significantly across development [44] are enriched for similar neuronal and cytoskeletal GO terms (e.g. locomotion, microtubule cytoskeleton organization) (S10 Table) confirming the highly dynamic and complex splicing regulation of these functional classes of gene.

In summary, we find that genes with key roles in the neuromuscular system and in the cytoskeleton appear to be have more complex splicing regulation: they tend to have more dynamic changes in splicing across development and they are enriched for the presence of multiple binding sites for SFs of widely differing biological functions. We suggest that this may allow the subtle fine-tuning of neuronal functionality across development.

## Discussion

During development, there are extensive and highly regulated splicing changes. How are these AS events regulated? Is any AS event regulated by a single SF or do multiple SFs combine to regulate individual AS events? In this study, we studied four splicing factors (SFs) in *C*. *elegans* to try to determine their individual targets and in particular to examine whether there was any evidence that they cooperate to regulate splicing.

We used three pieces of data to examine how any individual SF affects splicing. We first used RNA-seq to examine how a loss-of-function mutation in each of the four SFs affects splicing patterns. We could confirm ~80% of the identified splice changes using RT-PCR and showed a strong overlap in the AS events affected in two different strains that each has a loss-of-function mutation in the SF MEC-8, suggesting that our data are of high quality. Overall, we found that <10% of cassette exon AS events were affected in any individual mutant showing that the effect of losing any single SF has specific and limited effects on splice patterns. The AS events that are affected by the loss of any individual SF are likely to be a mix of direct targets of the SF and indirect downstream effects. To distinguish between these, we used a second dataset–the *in vitro* binding specificities of each SF which we had measured previously [68]. We reasoned that AS events that change in any SF mutant that have binding motifs for the SF are likely to be direct targets whereas AS events that are affected by the activity of any SF but that have no discernable binding motif for that SF are likely to be indirect. This approach has been used by other groups and successfully identifies direct SF targets [27,30,71,72,95,96]. Finally, we used evolutionary signals to identify the SF binding motifs that are most likely to be functional—binding motifs for any SF that are present in 5 diverse *Caenorhabditis* species have been conserved across long evolutionary time periods and are thus likely to be important whereas those that turn over rapidly are less likely to be functionally significant.

We thus combined direct measurements of how loss of each SF affects splicing patterns, *in vitro* measurements of each SF’s binding specificity, and evolutionary signatures, to identify the direct targets of each of the four SFs. Surprisingly, we find that there are significant overlaps in the targets of the four SFs. We use RT-PCR to confirm that multiple transcripts are indeed affected by multiple SFs and that loss of function of multiple SFs has additive effects on individual AS events. Finally, at least in the case of EXC-7 and MEC-8, we show that these combinatorial effects on splicing are likely to be functionally significant—a loss of function mutation of either *mec-8* or *exc-7* alone has only weak phenotypic effects whereas mutation of both genes together results in more severe fitness defects, as well as reduced sensitivity to aldicarb that suggests more severe defects in synaptic transmission. We note that MEC-8 and EXC-7 have also been shown to physically interact [83], underlining the likely relevance of the combinatorial effects we observe on splicing. We also show, using a bichromatic splicing reporter, that these SFs can regulate splicing of exons (e.g. exons 17–18 in *unc-52*) in the same cells.

We also find that the positions and orientation of the SF motifs in any transcript indicate that SFs may combine to regulate AS events in two distinct ways. For example, EXC-7 and FOX-1/ASD-1 binding motifs in co-regulated targets tend to occur close together—indeed the closer the motifs sit, the more likely the binding motifs are to be conserved, suggesting that the proximity is functionally relevant. For other pairs of SF, the basis for the combinatorial interactions appears to be different, relying not on proximity of the binding sites but rather on their relative orientation. For example, EXC-7 and MEC-8 sites tend to have a conserved ordering around any target AS events: when EXC-7 sites are upstream of MEC-8 sites in *C*. *elegans*, this same ordering is frequently seen in all 4 other species although the precise spacing between the sites may vary considerably. We thus suggest that some SFs (such as ASD-1/FOX-1 and EXC-7) may combine to regulate individual AS events through binding to closely located binding motifs in the same transcript whereas others (e.g. EXC-7 and MEC-8) may interact in more complex spatial ways that are dependent on ordering rather than proximity. While we do not know the specific mechanisms by which these SFs combinatorially regulate AS events, we find that these SFs may act together through longer distances to regulate the same splice site, distinct from examples of cooperative binding that were found for other pairs of SFs [46,49,51].

Our data thus suggest that the splicing of many transcripts are regulated by multiple SFs. Intriguingly, this combinatorial regulation by multiple SFs particularly affects transcripts from genes that play key roles in the neuromuscular system. In particular genes that encode ligand-gated ion channels and the many key cytoskeletal components that are required for neuronal and muscle cell function are potentially regulated by multiple of the SFs we studied. We do not believe that this is due to the initial selection of the 4 SFs that we studied since we find a similar trend in a wider set of 16 known or predicted SFs. For genes with binding sites for 4 or more SFs, we also find an enrichment of GO terms associated with functions in the neuromuscular system (e.g. cytoskeletal protein binding, transporter activity). We thus suggest that genes with key roles in the neuromuscular system appear to have complex splicing regulation, and their splicing is regulated by many individual SFs. We also note that the genes with binding sites for multiple SFs are enriched for having dynamic regulation of their splicing patterns across development [44] and we suggest that this high complexity in the regulation of neuronal splicing genes may allow fine-tuning of neuronal functions across development.

In summary, we used a combination of RNA-seq and *in vitro* RNA-binding specificities to systematically identify likely direct AS targets for 4 *C*. *elegans* SFs. These data provide a resource that contributes to our understanding of how these SFs regulate AS of specific *C*. *elegans* genes. The identification of candidate AS events that may be co-regulated by several SFs also provides a starting point for future studies to look at the variety and intricacies of combinatorial splicing regulation in *C*. *elegans*.

## Materials and methods

### RNA isolation and library preparation

All *C*. *elegans* strains were provided by the CGC, which is funded by NIH Office of Research Infrastructure Programs (P40 OD010440), and maintained at 20°C on NGM plates with OP50 bacteria. PolyA+ RNA was isolated from synchronized L4 worms, synthesized into cDNA and prepped for RNA sequencing as previously described [44].

### Identification of differentially spliced exons

All RNA-seq files in this study are available from NCBI/SRA under accession #PRJNA412927.

Each sequenced sample yielded approximately 200 million, 100bp paired-end reads, which were mapped to the WS220 version of the *C*. *elegans* genome using the RNA-Seq unified mapper (RUM) program [97] at default parameters. Uniquely mapped reads that spanned exon-exon junctions, and were annotated by RUM as “high-quality junctions”, were used to calculate splice junction usage.

The database of AS events that we used to compare splice site usage was generated using a combination of two methods. First, we generated an exon isoform database for *C*. *elegans* using SpliceTrap v.0.90.1 [98] and transcript annotations from Ensembl. SpliceTrap generates this database by subdividing each transcript isoform into exon trios to query for alternative splicing of the middle exon. To supplement the SpliceTrap-generated database, we also created our own custom database of known and possible *C*. *elegans* AS events from WS220 exon-level annotations downloaded from BioMart, by using a similar method that had been used to create an AS database for *Drosophila* [99]. The non-redundant set of possible AS events from these two databases were used to query for AS differences between samples.

For comparisons of splice site usage, we tested all events that have at least a combined 10 reads from junctions corresponding to the two different splice forms. For cassette exon and mutually exclusive events, we excluded instances where there is a greater than 30-fold difference between the numbers of reads mapping to the two adjacent junctions.

For cassette exons, we calculated percent-spliced-in (PSI) values as

$$PSI=100\times \frac{\frac{1}{2}(C1A+AC2)}{\frac{1}{2}(C1A+AC2)+C1C2)},$$

with C1 and C2 representing constitutive exons, A representing the alternative exon, C1A and AC2 corresponding to the number of reads mapping across the adjacent junctions, and C1C2 to the number of reads mapping across the alternative junction.

For instances with alternative donor or acceptor splice sites at the flanking exons, we calculated PSI values by summing all the combinations of adjacent junctions, similar to previously described methods [100]:

$$PSI=100\times \frac{\frac{1}{2}(\sum C_{i}A+\sum AC_{j})}{\frac{1}{2}(\sum C_{i}A+\sum AC_{j})+\sum C_{i}C_{j}},$$

where C_i_ and C_j_ are detected alternative donor and acceptor splice sites for the C1A and AC2 junctions respectively.

We then used Fisher’s exact test (2-sided) to test for significant differences in junction ratios between wild-type N2 and mutant samples. To identify splice changes, we require these events to have an accompanying p-value < 0.05, and a change in percent inclusion of at least 15%.

The same method was used to identify splice changes in other types of AS events.

### Validation of splicing changes using semi-quantitative RT-PCR

For preparation of L4-staged samples, worms were synchronized using the bleaching method and an additional sorting step was done on a Union Biometrica COPAS worm sorter using an empirically determined window of TOF and EXT values that captures L4 worms. RNA was isolated using TRIzol reagent (Invitrogen) using standard RNA extraction protocols. After digestion with DNase I (Sigma), random nonamers (Sigma) were used to synthesize total RNA into cDNA using SuperScript III reverse transcriptase (Invitrogen). PCR amplification cycles used ranged from 27–35 cycles, and PSI values were estimated by densitometric analysis using ImageJ [101].

### RNAi (fitness assay)

We used a quantitative method to assay for overall fitness of worm populations as previously described [102,103]. L1 worms were collected after putting worms through an 11μm filter. Bacteria expressing dsRNA were grown in LB media (supplemented with 1mM Carbenicillin) overnight at 37°C. Cultures were then induced with 4mM IPTG at 37°C for 2h, then spun down and resuspended in the same volume of NGM media (supplemented with 1mM Carbenicillin and 4mM IPTG). Approximately 10 worms were dispensed into each well in a 96-well plate along with 65μl of resuspended bacteria. Worms were then grown in a 20°C shaking incubator at 200rpm. Worms were harvested after 4.5 days and the population of worms in each well was assayed using a COPAS worm sorter, recording the time-of-flight (TOF) and extinct (EXT) values of each worm. In each independent experiment (4 total), 2–3 technical replicates were plated, with at least one blank well separating each different condition.

GFP RNAi clones were used as a non-targeting control, with 16–24 technical replicates plated for each experiment. Population averages were taken across the technical replicates and used as a proxy value for fitness. For both the wild-type and mutant *exc-7* strain, the relative fitness of worms subjected to *mec-8* RNAi in each strain was calculated by normalizing the *mec-8* RNAi population size to the control GFP RNAi population size. The expectation value for fitness of the mutant strain subjected to *mec-8* RNAi was then calculated using a multiplicative model (relative fitness in the wild-type strain targeted with *mec-8* dsRNA x relative fitness in the *exc-7* mutant strain with non-targeting dsRNA). The differences in actual fitness of the mutant strain subjected to *mec-8* RNAi relative to expected was then calculated. As control, the differences in actual fitness of the mutant strain subjected to control GFP RNAi relative to expected was also calculated. The significance of difference in log ratio of actual and expected values between the *mec-8* RNAi and control RNAi conditions was then calculated using a student’s t-test.

The same method was used screen for RBP interactors in other mutant SF backgrounds—*asd-1(ok2299)*, *fox-1(e2643)* and *mec-8(u218)*/*mec-8(303)*. S6 Table lists the primer pairs used to target various genes encoding known or putative SFs in each of the 4 SF mutant strains. Interaction scores were calculated as the actual fitness score relative to expected. Similarly, an interaction score of 0 implies a maximal negative interaction and a score of 1 implies no genetic interaction.

Gene pairs with an average interaction score < 0.7 across independent experiments are listed in Table 5. Scores involving *mec-8* mutants were calculated as the average interaction score between the two *mec-8* mutants tested.

### RNAi (RT-PCR)

For each RNAi knockdown, approximately 50 L4 worms were grown on NGM plates (supplemented with 1mM IPTG and 1mM Carbenicillin) seeded with dsRNA-expressing bacteria. GFP-targeting dsRNA-expressing bacteria was used as a negative control. L4-staged progeny were then harvested using a COPAS worm sorter and RNA samples were obtained as described above.

### Finding conserved motifs

To search for binding motifs for each splicing factor, we looked for the occurrence of GCAUG, GCACA and U(A|G|U)(A|G)GUU for FOX-1/ASD-1, MEC-8 and EXC-7 respectively. For each AS exon, these motifs were searched in the alternative exon, flanking introns and flanking constitutive exons at regions up to 300nt proximal to each splice site. To filter for conserved motifs, we downloaded the 7-way multiple alignment of *Caenorhabditis* genomes from the UCSC Genome Browser database (http://genome.ucsc.edu/) [82] and used alignment data from the 5 *Elegans* group species—*C*. *elegans*, *C*. *briggsae*, *C*. *remanei*, *C*. *sp*.*11* and *C*. *brenneri*. Motifs were first searched in the *C*. *elegans* sequence and then, using the UCSC multiple alignment, we consider a motif as conserved if it is present in 2 or more other *Elegans* group species within 25nt of the motif position in *C*. *elegans*.

The same method was used to identify conserved motifs corresponding to other known or putative SFs. All binding motifs (directly determined as well as inferred) were taken from the CISBP-RNA database [68]. The binding motifs for each SF that were used to find potential binding sites in this paper are listed in S4 Table.

### Enrichment of motifs

To test for enrichment of binding motifs, a set of exons that are alternatively spliced in our data at the L4 stage (5 to 95 PSI) was used as a background control to the set of differentially spliced exons. From this background, we then picked a random set of non-SF-regulated cassette exons with a matched N2/wild-type PSI distribution to the set of differentially spliced exons. Enrichment of each motif was tested using Fisher’s Exact Test. The final background/control value presented was the average value taken from 100 randomized samples. For differentially spliced exons in *fox-1*, *asd-1* and *exc-7* mutants, as well as the matched control sets, we considered each event to have a conserved motif if at least one is present within flanking introns. For differentially spliced exons in *mec-8* mutants and their matched control sets, we also considered conserved motifs in the alternative exons in addition to the flanking introns.

To calculate the number of exons with potential binding motifs for each SF, we looked for conserved motifs in the same manner among the set of exons that are alternatively spliced in our data at the L4 stage (5 to 95 PSI).

### Co-occurrence of motifs

For testing co-occurrence of motifs, we considered different motifs to co-occur if one or more of each motif is present within either flanking introns in the same AS event. We then used a cumulative hypergeometric distribution function to test for significance of co-occurrence [104,105]. We used a multiplicative model to determine expected co-occurrence values, where the expected proportion of co-occurring motifs are calculated based on the observed proportions of AS events with one or more of either motif. We used the same method for finding conserved motifs for each SF as described above. While dinucleotide content could over-estimate rates of motif co-occurrences—e.g. occurrences of two motif may not be independent due to biases in GC content [106]—we find that, while there are differences in dinucleotide composition in regions where we find co-occurring SF motifs (S6 Fig), these differences are relatively small (<1.2 fold), and should not greatly affect our conclusions.

The R GOstats package (v.2.32.0) [107] was used to look for GO term enrichment among the set of genes with motifs co-occurring at sites surrounding one or more of its cassette exons. A background set of AS events with PSI values between 1% and 99% at the L4 stage was used for each comparison. For analyses involving genes that contain temporally regulated AS events, the sets of genes that were previously identified to be differentially spliced across development [44] were used. This includes genes that were identified using both RNA-seq and microarray methods (listed in S2 and S3 Tables in Ramani *et al*.) [44]. Significance of overlap between the set of genes with multiple SF motifs and the aforementioned set of genes that are differentially spliced across development was calculated using a one-sided hypergeometric test.

To normalize for differing total intron lengths between genes with specific GO terms and the set of background genes, we picked a random set of genes from the background set with a similar intron length distribution to each set of genes with a specific enriched GO term. Each random sample was taken from a background set of genes that have been annotated with a GO term (MF or BP depending on the GO term tested) but not annotated with the specific GO term or any of its child terms. The proportion of genes with co-occurring motifs at one or more AS event was then calculated for both the random sample and the gene set with the specific GO term or its child terms. The final values presented for the control set was taken from the total of 1000 randomized samples. All GO annotations were taken from the GO.db (v.3.0.0) and org.Ce.eg.db (v.3.0.0) packages in R.

### Distances between motifs

For analyzing distances between pairs of SF binding motifs, we first looked for motifs at intronic regions proximal to splice sites. We split up each intron to denote two distinct intronic regions as either proximal to the 5’ or 3’ splice site. As previously, we restricted our analysis to regions up to 300nt proximal to each splice site.

We then carried out the distance analysis on each intronic region (5’ and 3’ ends of upstream introns and the 5’ and 3’ ends of downstream introns), where the distance between motifs was represented as the spacing between the end of the upstream motif and the start of the downstream motif. For the conservation analysis, we queried for motifs in other species at sequences that aligned with each of the respective intronic regions. For cases in which multiple instances of the same motif are present in an intronic region, we used the instance of that motif that is closest in distance to the second distinct motif to calculate the spacing between motifs in *C*. *elegans*. For spacing comparisons in other species, if multiple possible motif combinations exist, we used the comparisons of pairs of distinct motifs that result in a spacing distance that is most similar to the spacing distance in *C*. *elegans*. For plotting distribution of conserved spacing between motifs, we define a spacing between motifs as conserved if the motifs are present in 2 or more other *Caenorhabditis* species besides *C*. *elegans*, and if the length of the spacing in those other species are within 20% of the spacing observed in *C*. *elegans*. For plotting positions of co-occurring EXC-7 and MEC-8 motifs, we considered all instances in which both motifs are present (anywhere) in either introns, and in which these motifs are also present in 1 or more other species within a 25nt sliding window.

### Aldicarb assay

The aldicarb acute assays were all performed in liquid M9 in 96-well plates. Wild-type and mutant L4 worms were first grown on plates seeded with bacteria expressing dsRNA targeting either *exc-7* or GFP as a negative control. After 2 days, L1 progeny from these plates were isolated by filtration and used for the drug assay. Approximately 100 L1 worms were added to each well containing 2mM aldicarb or DMSO in M9 (total volume of 200μl). After 3h, the movement of worms after aldicarb treatment was compared to the movement of worms in the no drug control. Worms were scored as ‘moving’ if they exhibit wild-type-like movement similar to those in the control. 4–6 wells of worms were scored for their responses to aldicarb across 4 biological replicates.

## Supporting information

S1 Fig Exons that are differentially spliced in *mec-8* mutants are enriched for instances of increased exon inclusion. Loss of each splicing factor results in both increased inclusion and increased skipping of exons in the mutants. For each mutant strain, the proportions of exon skipping (both single exon as well as multiple exons) events that show either increased exon inclusion or increased exon skipping are illustrated. The significance of differences between both cases of exon splicing changes was calculated using a binomial test (two-sided).
(TIF)

S2 Fig Enrichment of shared AS changes between SF mutants. The overlap of differentially spliced exons identified in various mutants was compared to that expected from a background of L4 AS events. Numbers indicate the fold difference in overlap over expected, and the colours of the borders illustrate the significance of enrichment using a one-tailed hypergeometric test.
(TIF)

S3 Fig Binding motifs for pairs of SFs are found in close proximity in introns flanking alternatively spliced exons. (A-B) Conservation of spacing between these motifs are biased towards smaller distances for co-occurring SF binding motifs for EXC-7 and SUP-12, and for FOX-1/ASD-1 and EXC-7. Pairs of motifs with conserved spacing tend to be found closer together. Conservation of spacing between motifs was defined as spacing between motifs that are also present in 2 or more other *Caenorhabditis* species besides *C*. *elegans* at a distance +/- 20% of the distance in *C*. *elegans*.
(TIF)

S4 Fig Locations of co-occurring EXC-7 and MEC-8 motifs at introns that flank alternative exons. Each instance of an EXC-7 and a MEC-8 motif co-occurring around the same cassette exon is represented by a dotted line connected to an orange (EXC-7) and a blue (MEC-8) point. The position of each point along the x-axis represents the relative position of the motif proximal to each splice sites. Only motifs that lie within 300nt proximal to each splice site were plotted.
(TIF)

S5 Fig Binding motifs for multiple SFs co-occur at introns flanking alternative exons of genes with neuronal functions. Examples of co-occurring motifs that are conserved at introns flanking alternatively spliced genes. Sequence alignments were taken from the UCSC Genome Browser. Only motifs that are present in introns and within 300nt of each splice site are illustrated. Conserved binding motifs for various SFs are highlighted in yellow. M8 = MEC-8, T1 = TIAR-1/TIAR-2/TIAR-3, S12 = SUP-12, S49 = SAP-49, S26 = SUP26, E7 = EXC-7, F1 = ASD-1/FOX-1, U75 = UNC-75, E1 = ETR-1.
(TIF)

S6 Fig Dinucleotide content of intronic regions flanking alternative exons. The dinucleotide composition of introns that contain an EXC-7 but not a FOX-1/ASD-1 motif (blue; 124 instances) or introns that contain co-occurring EXC-7 and FOX-1/ASD-1 motifs (grey; 96 instances) are illustrated. Significance of differences in dinucleotide content between the two sets was calculated using a Mann-Whitney U Test (* p < 0.05, ** p < 0.01).
(TIF)

S7 Fig *mec-8* mutants show disorganized body wall muscle fibres. Muscle organization in L4 RW1596 (stEx30 [*myo*-*3*::gfp, rol-6(su1006)]) [108] transgenic worms were compared to RW1596 worms that were crossed into a *mec-8(u303)* mutant background.
(TIF)

S1 Table Total AS events detected and profiled at the L4 larval stage in N2. (XLSX)

S2 Table Exons that are differentially included in one or more SF mutant. (XLSX)

S3 Table Proportion of affected AS event with SF binding motifs at surrounding splice sites. (XLSX)

S4 Table Binding motifs for known and putative SFs used in this study. (XLSX)

S5 Table Co-occurrence of known and putative SF binding motifs at sites surrounding cassette exons. (XLSX)

S6 Table Genetic mutants and RNAi clones used in fitness screen. (XLSX)

S7 Table Functional annotation of genes with co-occurring SF motifs at sites surrounding alternative exons. (XLSX)

S8 Table Enrichment of GO terms among genes with co-occurring SF binding motifs at sites surrounding alternative exons. (XLSX)

S9 Table Enrichment of GO terms among genes with co-occurring SF binding motifs at introns flanking alternative exon. (XLSX)

S10 Table Enrichment of GO terms among genes with AS events that are differentially regulated across development. (XLSX)
