# Supplementary figures and images for: The combinatorial control of alternative splicing in C. elegans
Source: PLoS Genet. 2017 Nov 9;13(11):e1007033. doi: 10.1371/journal.pgen.1007033 (PMC5697891; doi:10.1371/journal.pgen.1007033)

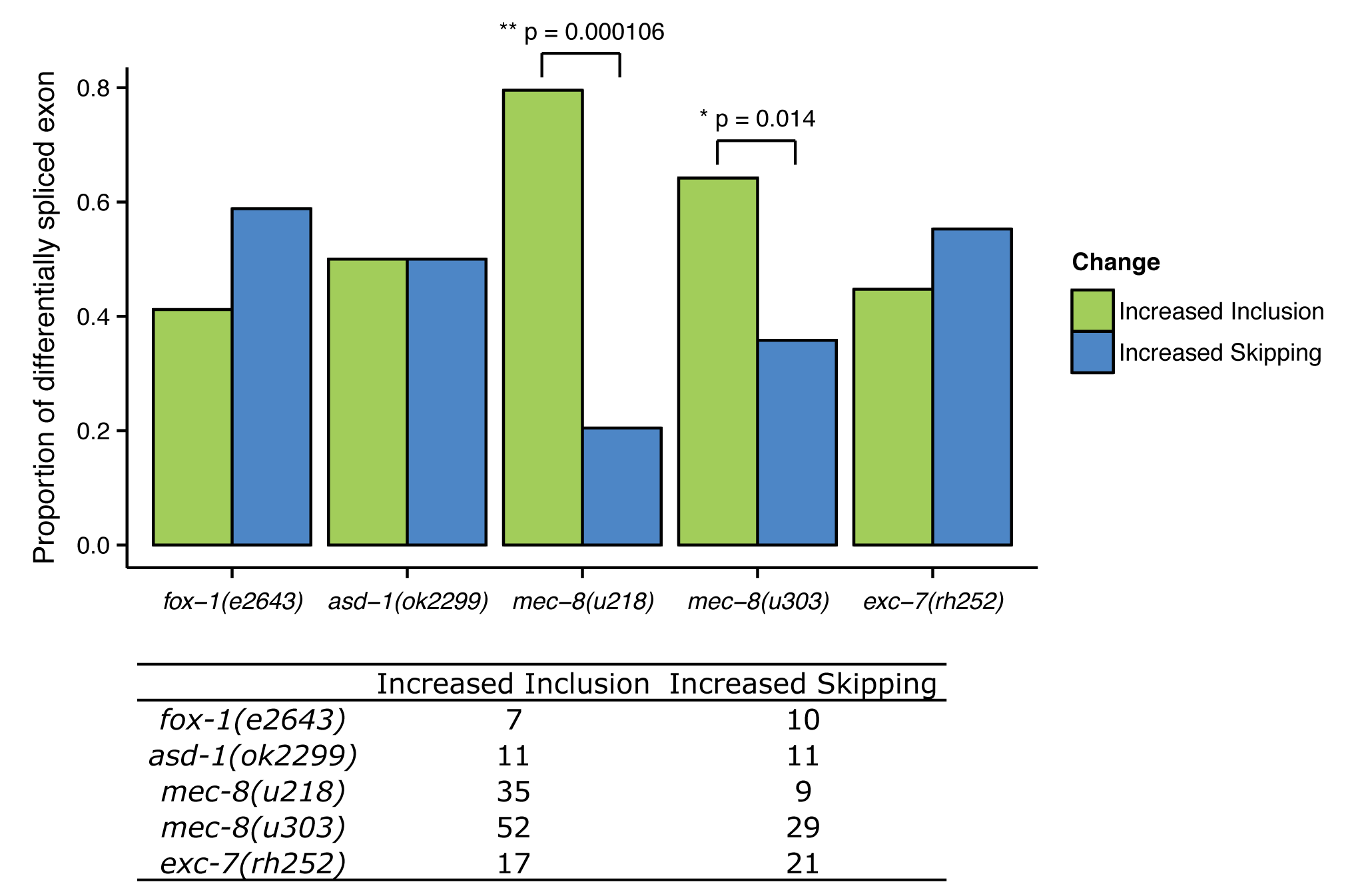

Supplement: S1 Fig — Loss of each splicing factor results in both increased inclusion and increased skipping of exons in the mutants. For each mutant strain, the proportions of exon skipping (both single exon as well as multiple exons) events that show either increased exon inclusion or increased exon skipping are illustrated. The significance of differences between both cases of exon splicing changes was calculated using a binomial test (two-sided). (TIF) [file pgen.1007033.s001.tif]

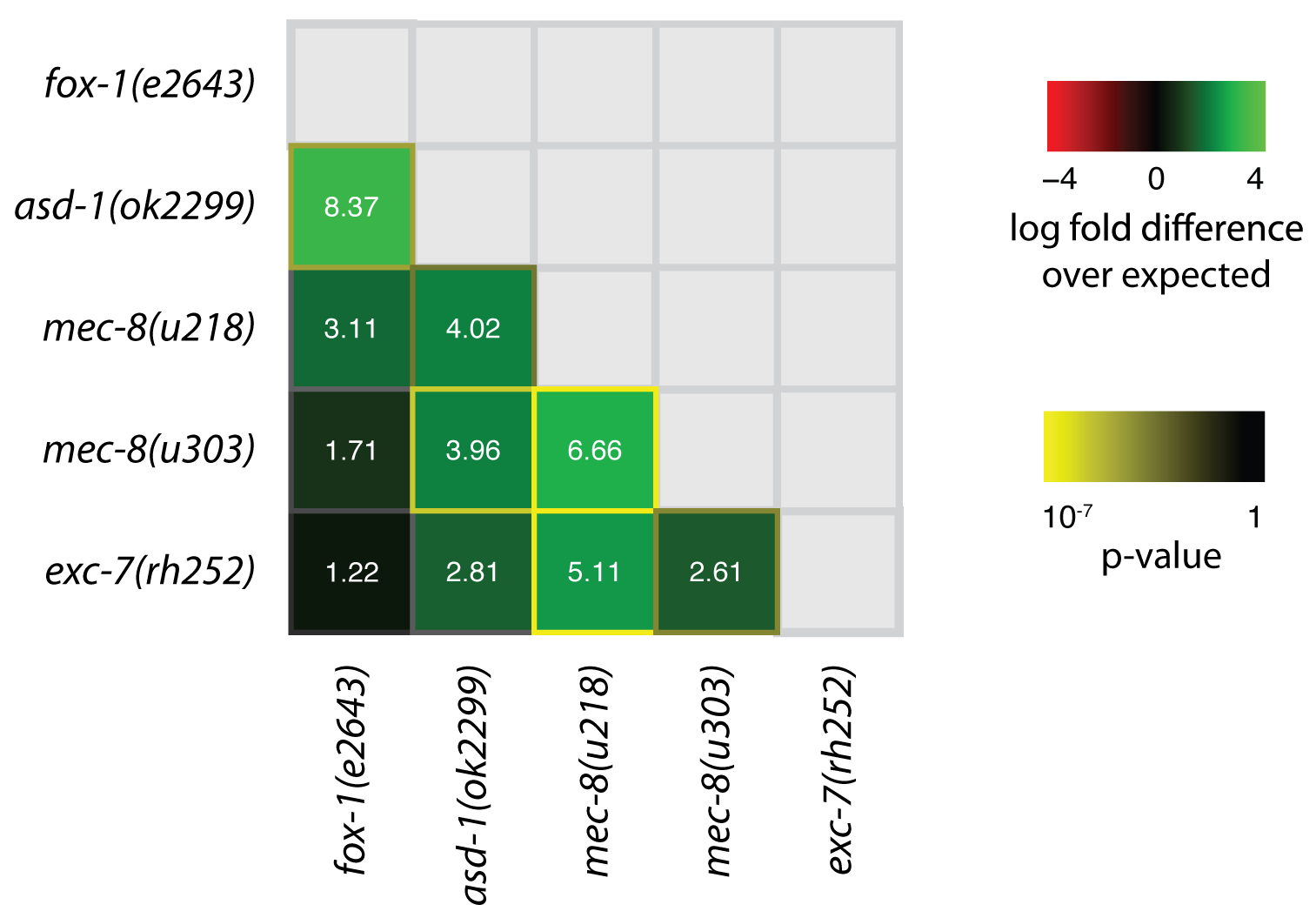

Supplement: S2 Fig — The overlap of differentially spliced exons identified in various mutants was compared to that expected from a background of L4 AS events. Numbers indicate the fold difference in overlap over expected, and the colours of the borders illustrate the significance of enrichment using a one-tailed hypergeometric test. (TIF) [file pgen.1007033.s002.tif]

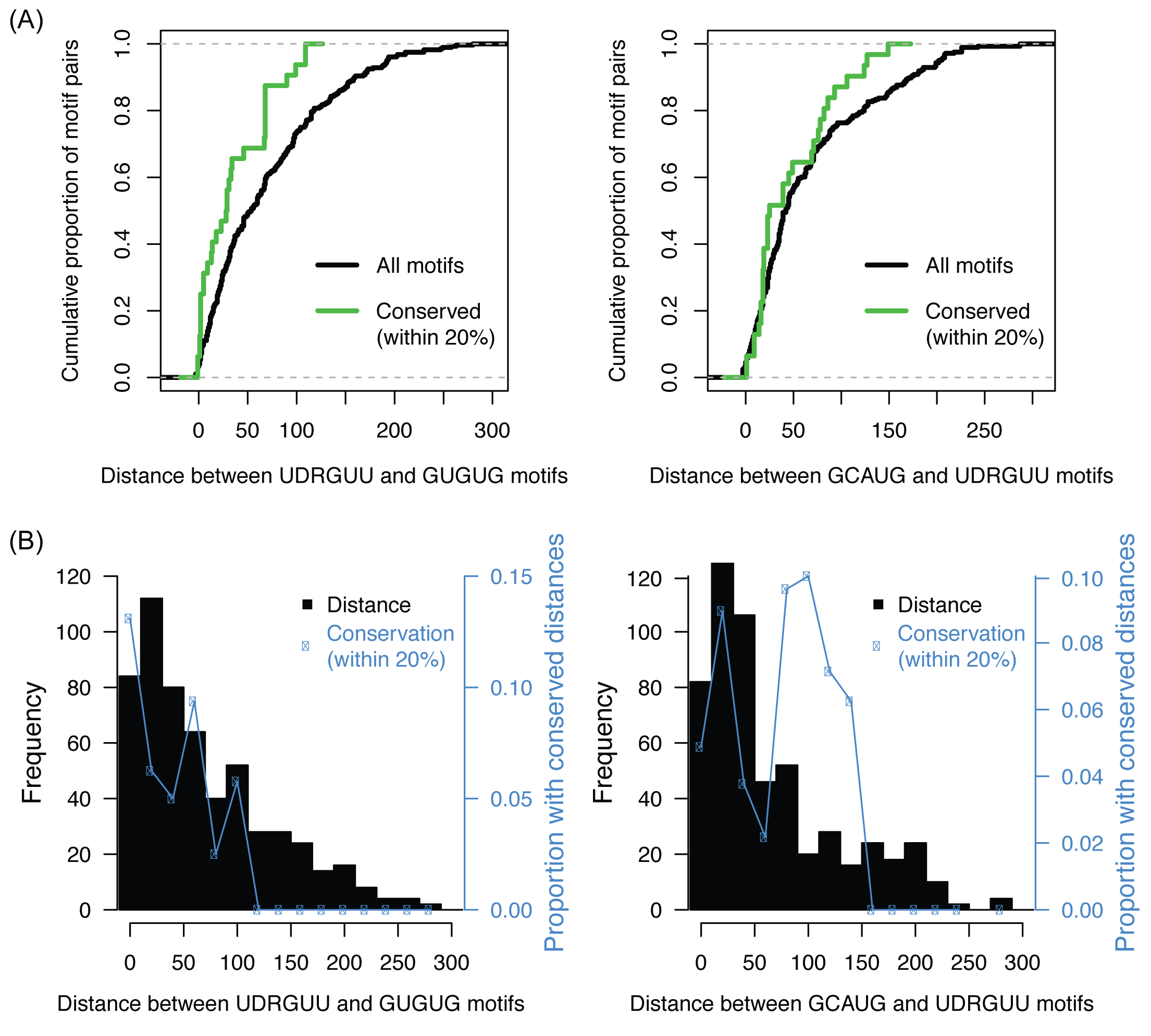

Supplement: S3 Fig — (A-B) Conservation of spacing between these motifs are biased towards smaller distances for co-occurring SF binding motifs for EXC-7 and SUP-12, and for FOX-1/ASD-1 and EXC-7. Pairs of motifs with conserved spacing tend to be found closer together. Conservation of spacing between motifs was defined as spacing between motifs that are also present in 2 or more other Caenorhabditis species besides C. elegans at a distance +/- 20% of the distance in C. elegans. (TIF) [file pgen.1007033.s003.tif]

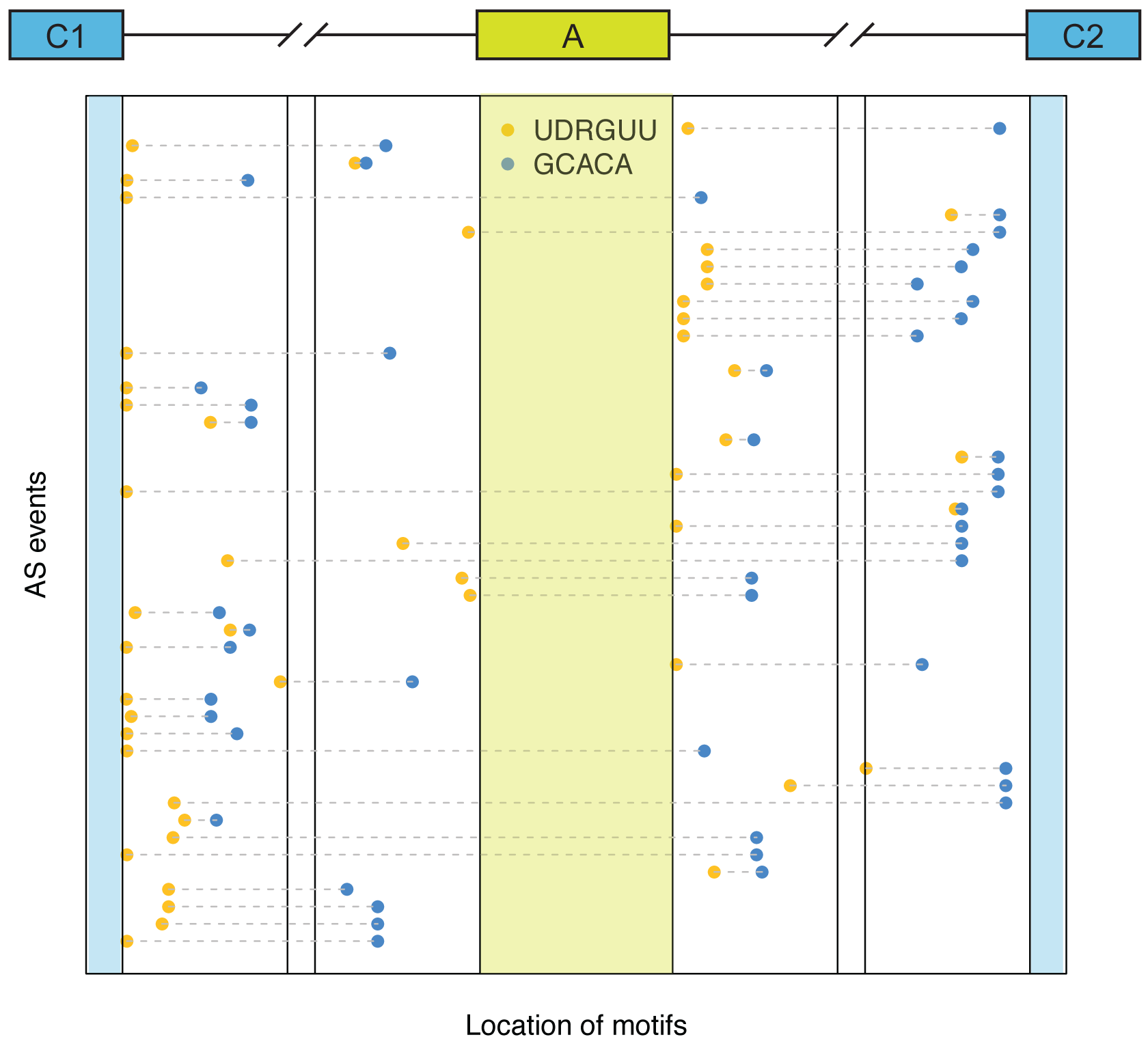

Supplement: S4 Fig — Each instance of an EXC-7 and a MEC-8 motif co-occurring around the same cassette exon is represented by a dotted line connected to an orange (EXC-7) and a blue (MEC-8) point. The position of each point along the x-axis represents the relative position of the motif proximal to each splice sites. Only motifs that lie within 300nt proximal to each splice site were plotted. (TIF) [file pgen.1007033.s004.tif]

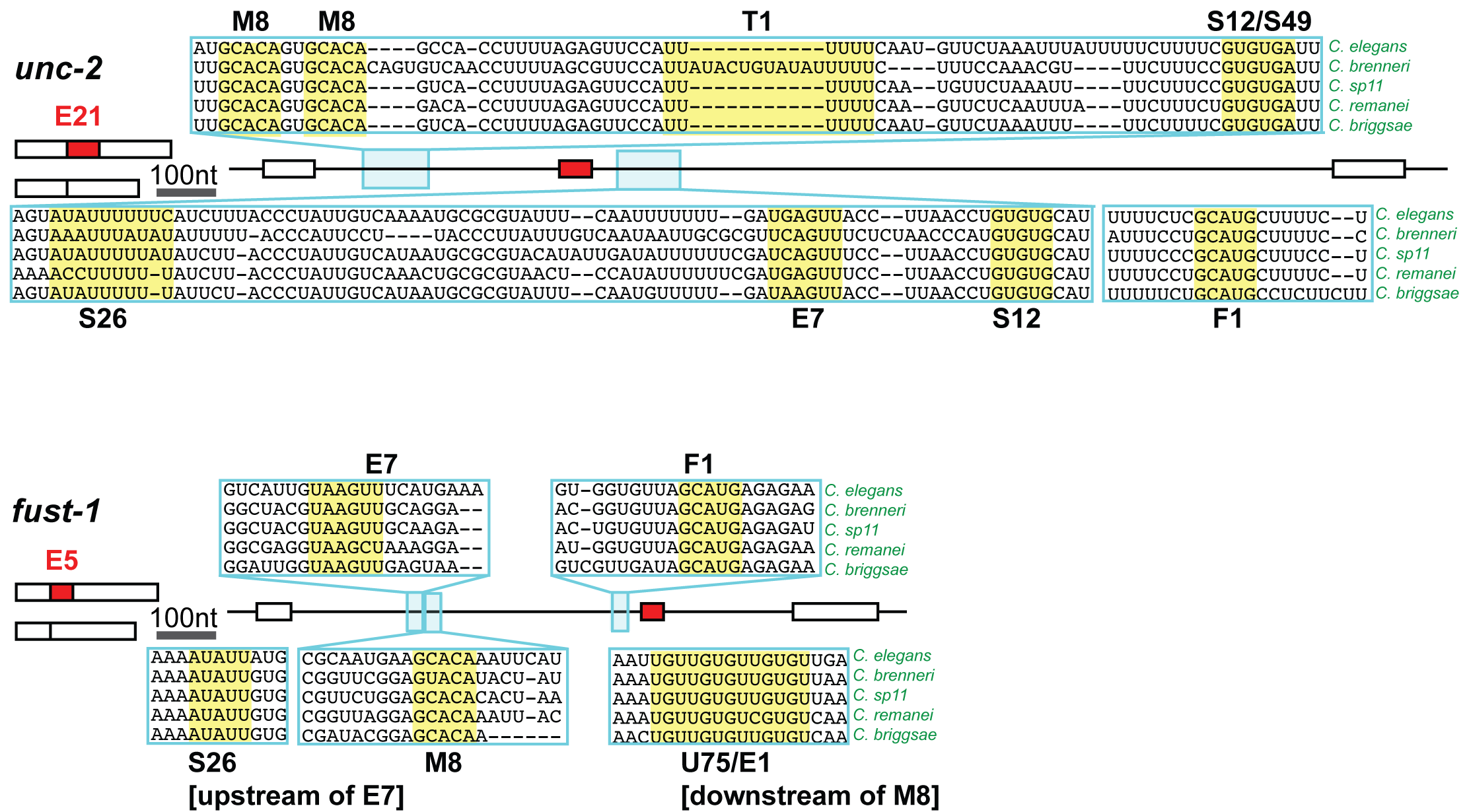

Supplement: S5 Fig — Examples of co-occurring motifs that are conserved at introns flanking alternatively spliced genes. Sequence alignments were taken from the UCSC Genome Browser. Only motifs that are present in introns and within 300nt of each splice site are illustrated. Conserved binding motifs for various SFs are highlighted in yellow. M8 = MEC-8, T1 = TIAR-1/TIAR-2/TIAR-3, S12 = SUP-12, S49 = SAP-49, S26 = SUP26, E7 = EXC-7, F1 = ASD-1/FOX-1, U75 = UNC-75, E1 = ETR-1. (TIF) [file pgen.1007033.s005.tif]

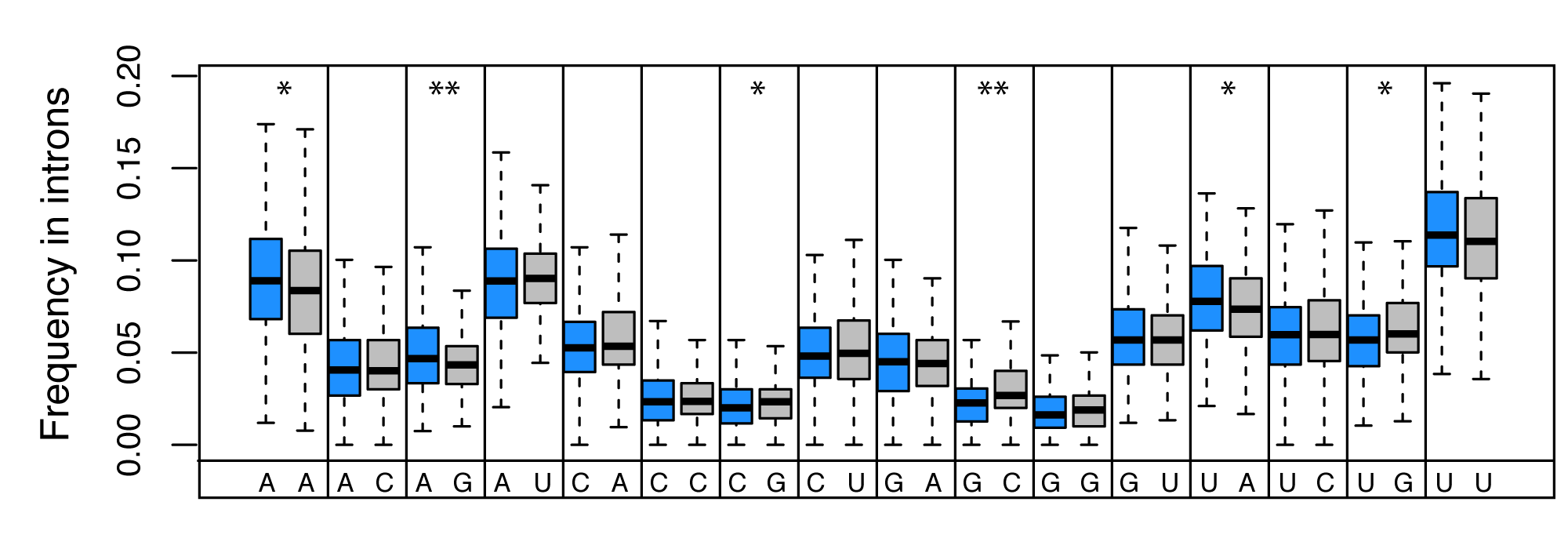

Supplement: S6 Fig — The dinucleotide composition of introns that contain an EXC-7 but not a FOX-1/ASD-1 motif (blue; 124 instances) or introns that contain co-occurring EXC-7 and FOX-1/ASD-1 motifs (grey; 96 instances) are illustrated. Significance of differences in dinucleotide content between the two sets was calculated using a Mann-Whitney U Test (* p < 0.05, ** p < 0.01). (TIF) [file pgen.1007033.s006.tif]

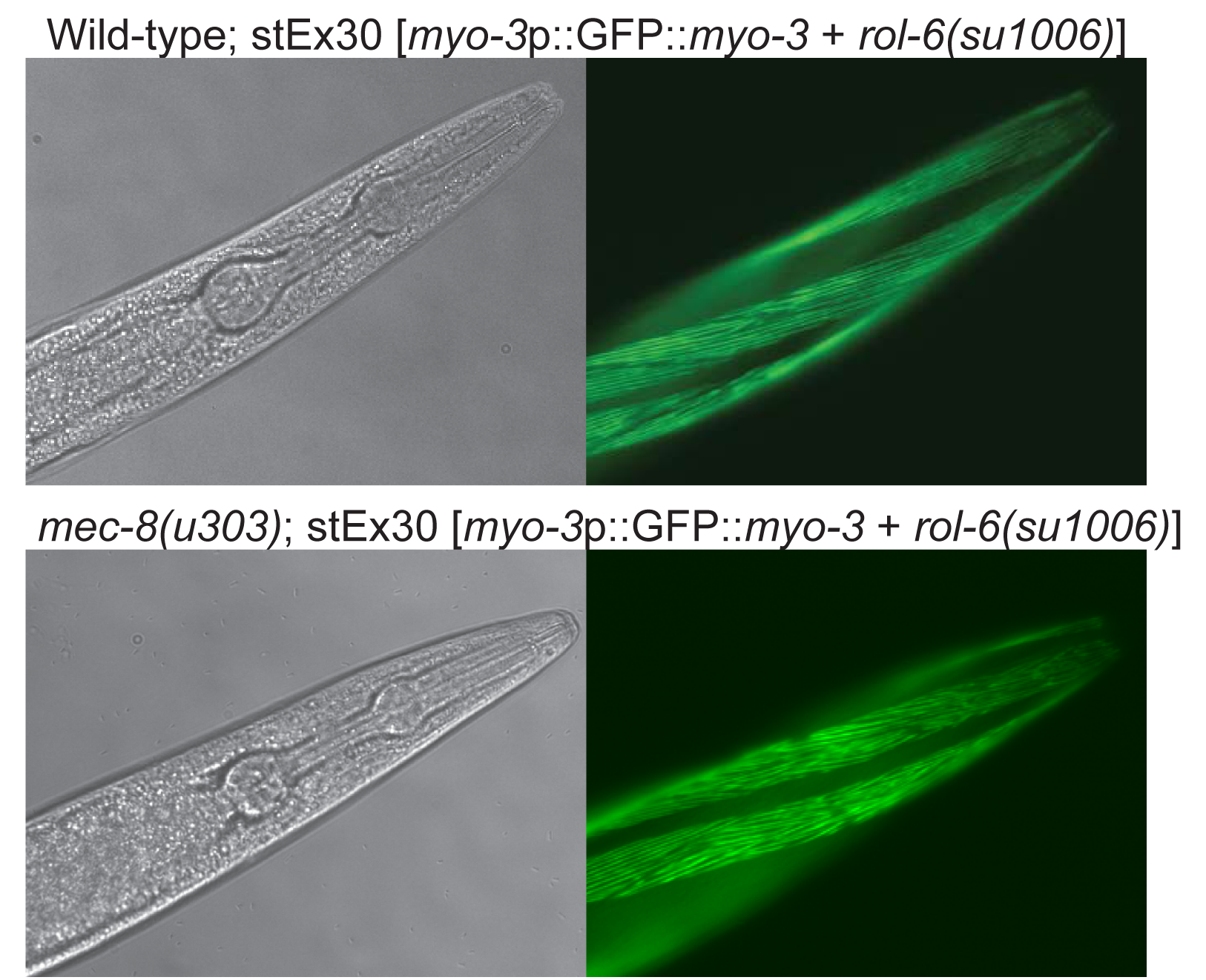

Supplement: S7 Fig — Muscle organization in L4 RW1596 (stEx30 [myo-3::gfp, rol-6(su1006)]) [108] transgenic worms were compared to RW1596 worms that were crossed into a mec-8(u303) mutant background. (TIF) [file pgen.1007033.s007.tif]
